# *N-*Oleoyl dopamine induces IL-10 via central nervous system TRPV1 and improves endotoxemia and sepsis outcomes

**DOI:** 10.1186/s12974-022-02485-z

**Published:** 2022-05-24

**Authors:** Jérémie Joffre, Erika Wong, Samira Lawton, Elliot Lloyd, Nina Nguyen, Fengyun Xu, Cristina Sempio, Lester Kobzik, Ivana Zlatanova, Mark Schumacher, Jost Klawitter, Hua Su, Katalin Rabl, Kevin Wilhelmsen, Che-Chung Yeh, Judith Hellman

**Affiliations:** 1 Department of Anesthesia and Perioperative Care, UCSF School of Medicine, 500 Parnassus Ave, Box 0648, San Francisco, CA 94143 USA; 2 Pediatric Critical Care Division UCSF Benioff Children’s Hospitals, San Francisco, CA 94158 USA; 3 Institute of Cognitive Science, CU Boulder, iC42 Integrated Solutions in Systems Biology, University of Colorado Denver, Aurora, CO 80045 USA; 4 Department of Pathology, Brigham and Women’s Hospital, Harvard Medical School, 75 Francis St, Boston, MA 02115 USA; 5 Cardiovascular Research Institute, UCSF School of Medicine, San Francisco, CA 94158 USA; 6 Division of Pain Medicine, UCSF School of Medicine, San Francisco, CA 94143 USA

**Keywords:** Endovanilloid, *N-*Oleoyl dopamine, TRPV1, Inflammation, Neuro-immune, Cytokines, IL-10, ALI

## Abstract

**Background:**

The transient receptor potential vanilloid 1 (TRPV1) participates in thermosensation and inflammatory pain, but its immunomodulatory mechanisms remain enigmatic. *N-*Oleoyl dopamine (OLDA), an endovanilloid and endocannabinoid, is a TRPV1 agonist that is produced in the central nervous system and the peripheral nervous system. We studied the anti-inflammatory effects and TRPV1-dependent mechanisms of OLDA in models of inflammation and sepsis.

**Methods:**

Mice were challenged intratracheally or intravenously with LPS, or intratracheally with *S. aureus* to induce pneumonia and sepsis, and then were treated intravenously with OLDA. Endpoints included plasma cytokines, leukocyte activation marker expression, mouse sepsis scores, lung histopathology, and bacterial counts. The role of TRPV1 in the effects of OLDA was determined using *Trpv1*^*−/−*^ mice, and mice with TRPV1 knockdown pan-neuronally, in peripheral nervous system neurons, or in myeloid cells. Circulating monocytes/macrophages were depleted using clodronate to determine their role in the anti-inflammatory effects of OLDA in endotoxemic mice. Levels of exogenous OLDA, and of endovanilloids and endocannabinoids, at baseline and in endotoxemic mice, were determined by LC–MS/MS.

**Results:**

OLDA administration caused an early anti-inflammatory response in endotoxemic and septic mice with high serum levels of IL-10 and decreased levels of pro-inflammatory cytokines. OLDA also reduced lung injury and improved mouse sepsis scores. Blood and lung bacterial counts were comparable between OLDA- and carrier-treated mice with *S. aureus* pneumonia. OLDA’s effects were reversed in mice with pan-neuronal TRPV1 knockdown, but not with TRPV1 knockdown in peripheral nervous system neurons or myeloid cells. Depletion of monocytes/macrophages reversed the IL-10 upregulation by OLDA in endotoxemic mice. Brain and blood levels of endovanilloids and endocannabinoids were increased in endotoxemic mice.

**Conclusions:**

OLDA has strong anti-inflammatory actions in mice with endotoxemia or *S. aureus* pneumonia. Prior studies focused on the role of peripheral nervous system TRPV1 in modulating inflammation and pneumonia. Our results suggest that TRPV1-expressing central nervous system neurons also regulate inflammatory responses to endotoxemia and infection. Our study reveals a neuro-immune reflex that during acute inflammation is engaged proximally by OLDA acting on neuronal TRPV1, and through a multicellular network that requires circulating monocytes/macrophages, leads to the systemic production of IL-10.

**Supplementary Information:**

The online version contains supplementary material available at 10.1186/s12974-022-02485-z.

## Background

The endovanilloid system is evolutionarily conserved from coelenterates to humans and includes members of the transient receptor potential vanilloid family (TRPV1–6) [1], endogenous lipid ligands, and enzymes involved in their biosynthesis and degradation [2, 3]. TRPVs are non-specific cation channels that are expressed by cells of the central and peripheral nervous systems (CNS and PNS, respectively), and by non-neuronal cells, including T-cells, microglia, astrocytes, microvascular endothelial cells, arteriolar smooth muscle cells and epithelial cells [4–11]. TRPVs are believed to play roles in cell development and growth, reproduction, pain, and behavior [12, 13]. Endovanilloids activate TRPVs and are derived from arachidonic acid precursors present in lipid membranes [14].

Two potent endovanilloids, *N-*arachidonoyl dopamine (NADA) and *N-*oleoyl dopamine (OLDA), are active at TRPV1 and cannabinoid receptors (CBRs) and thus are considered to be endovanilloids and endocannabinoids [15–23]. NADA and OLDA are acyl-dopamines that are produced in the CNS and PNS, and are putatively involved in neuronal functions and plasticity, and in pain pathobiology [11, 15, 16, 24–36]. Vanilloid and cannabinoid ligands have been reported to have immunomodulatory effects [4, 37, 38]. Although studies suggest that the endovanilloid and endocannabinoid systems may regulate immune functions, there is a minimal information about which cells and signaling mechanisms mediate the immunological effects of vanilloids and cannabinoids under inflammatory conditions.

Sepsis, which is defined as life-threatening organ dysfunction caused by a dysregulated host response to infection, is a leading cause of death worldwide, accounting for approximately 8 million deaths each year [39, 40]. We previously reported that exogenously administered NADA exerts strong TRPV1-dependent anti-inflammatory effects early during endotoxemia or polymicrobial abdominal sepsis induced by cecal ligation and puncture (CLP) [37]. Interplay between the immune and nervous systems has been postulated in the past, and recent studies have begun to unravel the cellular and molecular components of neuro-immune interactions [41, 42]. Notably, we have found that TRPV1 expressed outside of the myeloid compartment mediates NADA’s systemic anti-inflammatory effects [37]. We hypothesized, based on the high levels of TRPV1 expression by neurons, that acyl-dopamines like NADA and OLDA exert their anti-inflammatory effects via neuronal TRPV1. We also speculated that OLDA might have similar anti-inflammatory actions as NADA.

In the current studies, we tested the effects and cellular and molecular mechanisms of exogenously administered OLDA on systemic inflammation and on clinical outcomes in murine models of endotoxemia and bacterial sepsis induced by *S. aureus* pneumonia. We also investigated the endogenous regulation of OLDA and other endovanilloids and endocannabinoids in endotoxemic mice. We found that OLDA administration leads to strong and sustained anti-inflammatory effects and improves functional outcomes, including lung injury and mouse sepsis scores, in endotoxemic mice and in mice with *S. aureus* pneumonia. In endotoxemic mice, OLDA potently upregulates the production of IL-10 by peripheral monocytes/macrophages, but it does so indirectly via its activity at TRPV1 expressed by neurons. Our study uncovers a novel neuro-immune network that is engaged by the activity of OLDA at neuronal TRPV1 and leads downstream to upregulated systemic production of IL-10 mediated by peripheral monocytes/macrophages.

## Methods

### Animals

8- to 13-week male and female mice were utilized for experiments. The UCSF Institutional Animal Care and Use Committee approved all animal studies. Experiments were performed in accordance with the Public Health Service Policy on the Humane Care and Use of Laboratory Animals. C57BL/6J (wild-type), B6.129×1-*Trpv1tm1Jul*/J (*Trpv1*^−/−^), B6.129P2-Lyz2tm1(cre)Ifo/J (Lysm-Cre), STOCK Tg(Actl6b-Cre)4092Jiwu/J (BAF53b-Cre), and B6.129P2-Aviltm2(cre)Fawa/J (Advillin-Cre) were purchased from Jackson Laboratory, and were maintained in breeding colonies in our animal facilities. The *Trpv1*^lox/lox^ line was generated by Cyagen Biosciences Inc. (Santa Clara, CA) at our request. Briefly, to create a conditional knockout allele in the mouse TRPV1 genomic locus, we introduced 34 base pair LoxP sites on either side of exon 13 of the gene, which includes two transmembrane domains. In the absence of this exon, the subsequent coding region will be out of the frame, and the locus will not produce a functional TRPV1 protein. The homology sequences and the insertion of LoxP sites of the targeting construct were derived from BAC clones from the C57BL/6 genomic DNA library by PCR, which was cloned into pUC19 vector. Two gRNAs were designed to ensure that the cuts will include both loxP sites and exon 12 (5ʹ arm side sequence 5ʹ-GTTGAGTGGCTTTCCTGCTG-3ʹ; 3ʹ arm side sequence 5ʹ-AGGGTTGCATGAGCCCCTGT-3ʹ). The gRNAs, the donor vector containing loxP sites, and Cas9 mRNA were co-injected into fertilized mouse eggs to generate targeted knockout offspring through CRISPR/CAS9 mediated homologous recombination. PCR identified F0 founder mice, and the correct integration and sequences of both loxP sites were further validated by sequence analysis of PCR products from genomic DNA. *LysM-Cre/Trpv1*^*lox/lox*^, *BAF53b-Cre/TRPV1*^*lox/lox*^, *Advillin-Cre/Trpv1*^*lox/lox*^ were generated in our animal facilities. For each experiment we used age and sex-matched appropriate littermates as controls.

### Models of endotoxemia, LPS-induced acute lung injury, and *S. aureus* pneumonia and sepsis

Non-infectious acute inflammation was induced in wild-type and genetically modified mice by intravenous (i.v.) or intratracheal (i.t.) challenge with LPS (1, 3 or 5 mg/kg in 5 μl vehicle/g of body weight, LPS-*E. coli* O111:B4, Sigma-Aldrich) or the same volume of 0.9% saline (vehicle for LPS). *S. aureus* pneumonia was induced by direct i.t. injection of *S. aureus* (Newman strain, ATCC) diluted in saline at an estimated dose of 1 × 10^7^ colony-forming unit (CFU)/25 g of body weight. Mice with *S. aureus* infections were treated intraperitoneally (i.p.) with vancomycin (10 mg/kg) or an equivalent volume of diluent (i.p.) every 12 h. All mice had free access to food and water. Core temperatures were monitored at intervals (Thermalert TH-5, Physitemp^®^). The illness severity was assessed by measuring the murine sepsis score (MSS) [43] by a third-party investigator that was blinded to the experimental conditions.

### Treatment with OLDA

Stock solutions of OLDA dissolved in ethanol were purchased (Cayman Chemical). The ethanol was evaporated, and then OLDA was diluted in Tween 20 (5% final concentration) in PBS. OLDA (5 or 10 mg/kg, i.v. in a volume of 4 ul/g of body weight) or an equivalent volume of the control, 5% Tween 20 in PBS (vehicle), was administered to mice immediately or 2 h after LPS injection. OLDA and vehicle were prepared for experiments on the day of their administration to mice using sterile procedures.

### Monocyte depletion

Monocytes were depleted by treating mice with Clodronate liposomes (10 μl/g, i.v.; Formumax Inc.) 20 h prior to experimentation [37]. Control mice were treated with neutral liposomes (10 μl/g, i.v.; Formumax Inc.). Depletion efficacies were checked by performing flow cytometry on cells from multiple compartments. Treatment with Clodronate liposomes led to reductions in total monocytes of approximately 65% in the blood, 65% in the bone marrow and 60% in the spleen, and to reductions in Ly6C hi monocytes of 90% in the blood, 80% in the Bone marrow, and 65% in the spleen (data not shown).

### Splenectomy procedure

Under isoflurane anesthesia, we performed left lateral laparotomy, exteriorized the spleen, and ligated its vascular pedicle twice before splenectomy. The peritoneum was closed using sutures with 6-0 nylon thread, and the skin closed with wound clips. For sham surgery, the left lateral laparotomy was performed and we momentarily exteriorized the spleen before returning it to its position and closing the incision. Buprenorphine was used as an analgesic for the first 24 h. We waited 3 weeks post-splenectomy before experimentation.

### Hot plate test

We tested the sensitivity of mice to heat as a phenotypic test for neuronal TRPV1 knockdown using a conventional hot plate test (apparatus IITC Inc. Model 39) set to a temperature of 55 °C (± 0.2 °C). Mice were individually placed on the aluminum surface bordered by a transparent square enclosure. The latency to response is recorded when the mouse elicits a nocifensive behavior to the heat (e.g., hind paw withdrawal, licking, or jump). In case of absence of response, we used a 30-s cut-off time.

### Histology

Lung tissue was collected at the time of euthanasia and fixed with 4% paraformaldehyde, then underwent embedding, serial 5-μm sections, and hematoxylin–eosin staining by an outside company (HistoWiz, Inc.). They were then anonymized for blind analysis. Two sections per animal were randomly selected, and each section was divided into six areas to be quantified. Before quantification, pictures were anonymized by a third investigator. For lung sections, endothelial injury, epithelial injury, intra-alveolar hemorrhage, alveolar edema, and neutrophil infiltration were blindly scored using a semi-quantitative scale ranging from 0 to 4 [44, 45]. Neutrophil infiltration was blindly double-checked by an experienced pathologist. Lung condensation was automatically quantified with Image J software and score from 0 (aeration > 55%) to 4 (aeration < 20%).

### Flow cytometry

Blood, bone marrow, spleen, and BAL fluid were collected after killing and labeled with several antibody mixes to explore innate and adaptive immune responses in distinct compartments. Cells were labeled with Pacific Blue-anti-Cd11b (M1/70.15), APC (blood) or PECy7 (spleen)-anti-Ly6G (1A8), APC-anti-CD19 (1D3), PercpCy5.5-anti-CD25 (PC61.5), PECy7–anti-Foxp3 (FJK-16s), FITC-anti-CD4 (GK1.5), AF700–anti-CD8 (53–6.7), PEcy5-anti-CD40 (1C10), PE- anti-CD69 (N418) and APC-anti-GFAP (GA5) from InVitrogen™*.* FITC-anti-CD3 (17A2), PE-anti-Ly6C (HK1.4) and APC-anti-MHC II (M5/114.15.2) from BioLegend™. BV605-anti-Nk1.1 (PK136), Amcyan-anti-B220 (RA3-6B2), Percp-anti-CD45 (30F11), from BD Biosciences. PE-anti-CD11c (HL3), APC-anti CD44 (IM7) and ef450-CD62L (MEL-14) and AF700-anti-F4/80 (BM8) from eBiosciences™. For staining of circulating leukocytes, erythrocytes were lysed using B.D. fluorescence-activated cell sorter lysing solution (BD Biosciences™). For intranuclear staining, surface staining was performed before permeabilization using Foxp3 staining buffer kit (eBiosciences™) and intranuclear staining. Single-cell suspensions stained with fluorophore-conjugated antibodies and cell count estimation were acquired the day of killing using an LSRII Fortessa ™ (B.D. Biosciences) flow cytometer and analyzed with FlowJo software (Miltenyi^®^). Myeloid-derived suppressive cells were identified as follows: Monocytes were identified as CD11b+Ly6G−. Among them, classical monocytes or monocytes myeloid-derived suppressive cells (Mo-MDSCs) were Ly6Chigh and non-classical monocytes were Ly6Clow. Neutrophils were identified as CD11b+Ly6G+. Regulatory T (Tregs) cells were considered as CD3+CD4+CD25highFoxp3+ (forkhead box P3). B cells (CD19+ in spleen or B220+ in blood), CD4+, CD8+, NKT (CD3+NK1.1+) and NK cells (NK1.1+CD3−) lymphocyte subsets were also analyzed.

### Pharmacokinetic (PK) analysis of OLDA

For the PK studies, mice were injected with OLDA (10 mg/kg, i.v.) at *T* = 0 and blood was collected at *T* = 1, 2, 5, 15, 30, 60 min, and then at 2, 6 and 24 h to assess residual plasma concentration of OLDA (*n* = 4 mice per time point). Levels of OLDA were quantified by Cayman Chemicals Inc. using selective, multiple-reaction monitoring (MRM) Liquid Chromatography with tandem mass spectrometry (LC–MS/MS)-based method.

### Quantitation of endogenous lipids

For endogenous lipid assays, mice were injected with LPS (3 mg/kg i.v.) at *T* = 0, and brain and blood were collected at *T* = 10, 30 and 120 min and were immediately processed to preserve lipids. Plasmas were placed in glass vials. Endocannabinoids, endovanilloids, arachidonic acid, and prostanoids were quantified using a (LC–MS/MS)-based method. Aliquots of internal standard solution and blank 1:1 Acetonitrile (ACN): H_2_O (10 μl each) were added before gently mixing the tube contents. The diluted plasma was extracted with 60 μl of methyl tert-butyl ether (MtBE), thoroughly vortex-mixed for 30 s, then phase separated by centrifuging for 30 s at 16,100 rcf. The aqueous phases were frozen by placing the samples into − 80 °C freezer for approximately 2 min; then the organic phases were drawn off and placed in glass autosampler vials. The MtBE was dried under speed-vac centrifuge for 15 min, after which 50 μl 1:1 ACN:H_2_O was added back to the vial to reconstitute the contents for injection onto the instrument (Waters Acquity I-Class UPLC with Xevo, TQ-S micro MS/MS).

### Immunoassays

Blood samples were collected from the retro-orbital vein sinus of anesthetized mice into heparin-coated capillary tubes, or terminally by cardiac puncture into heparin-coated syringes Subsequently, the tubes were centrifuged, and the plasmas were removed and stored at − 80 °C until analysis. Brains were collected after killing and flushing the blood with cold PBS and immediately frozen in liquid nitrogen. Levels of IL-10, IL-6, and CCL2, were measured using Quantikine ELISA Kit^®^ (R&D Systems, Inc.), according to the appropriate dilution and following the manufacturer’s instructions. Multiplex assays were performed using the Procartaplex™ immunoassay technology (Thermo Fisher).

### Cell culture and in vitro experiments

Primary macrophages were derived from mouse bone marrow-derived cells (BMDM). Tibias and femurs of wild-type male mice were dissected, and their bone marrows were flushed out. Cells were incubated for 7 days at 37 °C, in humidified, 5% CO_2_ in a solution of RPMI 1640 medium, 20% neonatal calf serum, and 20% L929-conditioned medium, which is rich in macrophage-colony-stimulating factor. Primary human lung microvascular endothelial cells (HMVEC) and astrocytes from male and female cadavers (Lonza, Basel, Switzerland) were used at passage 3–6. HMVEC or astrocytes were cultured at 37 °C, 5% CO_2_ in appropriate tissue culture medium to 90–100% confluence before treatment. They were then simultaneously treated with *E. coli* 0111:B4 LPS (1 μg/ml; Sigma-Aldrich, St. Louis, MO) and test agents (e.g., OLDA).

### Measurement of intracellular calcium

Calcium imaging was performed to assess capsaicin-induced activation of DRG neurons from *Advillin-Cre*^+/*−*^*/Trpv1*^*lox/lox*^ and *Advillin-Cre*^*−/−*^*/Trpv1*^*lox/lox*^ mice. Mouse lumbar dorsal root ganglion cells (DRGs) were harvested from euthanized adult male mice. DRG neurons were cultured on coverslips overnight at 37 °C and subsequently loaded with calcium imaging buffer (Hank’s BSS with 20 mM HEPES, 100 U/ml penicillin and 0.1 mg/ml streptomycin, pH 7.4) containing 5 µM Fluo-4 (488/520 nm) for 45 min at 37 °C. DRG neurons were imaged on an Axiovert 200 microscope with video acquisition run by Zen 3.0 software (Carl Zeiss Light Microscopy, Germany) [46]. Solutions were applied at 2 ml/min perfusion rate at RT. Increases in intracellular calcium were observed following application of 1 µM capsaicin (10 s). Cell viability was determined by applying 25 mM KCl at the end of each recording.

### Quantitative real-time polymerase chain reaction (qPCR)

qPCR was performed after QIAzol Lysis Reagent (Qiagen) ribonucleic acid (RNA) extraction on an ABI Prism 7700 Sequence Detection System (Thermo Fisher Scientific, Inc.). Relative expression was calculated using the 2-delta-delta computed tomography (C.T.) method. All amplifications were TaqMan^®^ Real-time PCR Assays (Applied Biosystem TM, Thermo Fisher Scientific, Inc.).

### Statistics

Data were analyzed using two-tailed Mann–Whitney U tests. *P* values less than 0.05 were considered statistically significant. Statistics and graphical representations were performed using Prism 9.2 (Graph Pad Software Inc.). Results are reported as means ± SD. Group sizes are indicated in the figure legends for each experiment. Experiments were repeated at least twice. Results of animal studies are reported in concordance with the ARRIVE guidelines [47].

## Results

### Exogenous OLDA administration TRPV1-dependently reduces TLR2 and TLR4 agonist-induced systemic inflammation reduces the severity of endotoxemic shock

**Fig. 1:**
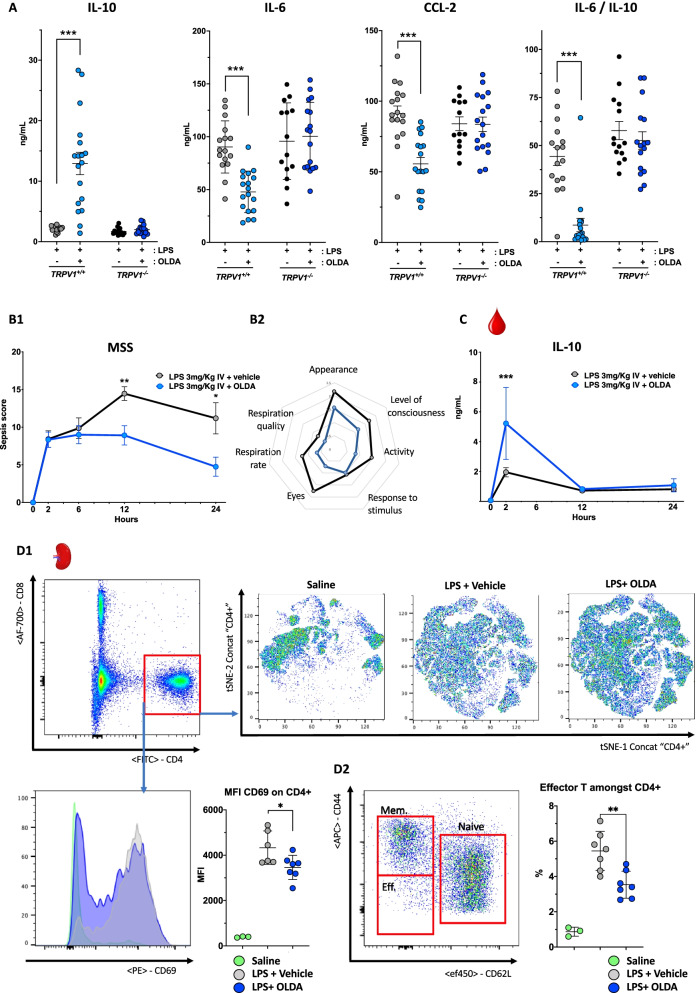
The anti-inflammatory and protective actions of exogenously administered OLDA in endotoxemic mice. **A** OLDA increases IL-10 and reduces circulating IL-6 and CCL2 levels in mice challenged with the TLR4 agonist LPS: Wild-type mice were challenged with LPS (1 mg/kg, i.v.) and immediately after with OLDA (10 mg/kg, i.v.) or vehicle (i.v.). IL-10, IL-6, and CCL2 were quantified in plasma at 2 h. Global TRPV1 genetic deficiency reversed the anti-inflammatory effects of OLDA (i.v.; 9- to 12-week male and female, *n* = 13–17/group). **B** OLDA reduces the severity of endotoxemic shock: Wild-type mice were treated with LPS (3 mg/kg, i.v.) and immediately thereafter with OLDA (10 mg/kg, i.v.) or vehicle (i.v.). **B1** Shows reduced Mouse Sepsis scores (MSS) in mice treated with OLDA. The diagram in **B2** shows a representation of average score for each item in the mouse sepsis score (from 0 (healthy) to 4 (very sick)) at 24 h (9- to 12-week male, *n* = 5–10 per group and time point, total *n* = 52). **C** OLDA induces an early rise in plasma IL-10: Wild-type mice were treated with LPS (3 mg/kg, i.v.) followed by OLDA (10 mg/kg, i.v.) or carrier (i.v.). Plasma IL-10 levels were significantly increased at 2 h, but not at 12 or 24 h in mice treated with OLDA (9- to 12-week male,* n* = 5 per group and time point, total *n* = 35). **D** OLDA administration reduced activation CD4+ spleen T cells in endotoxemic shock (9- to 12-week male, *n* = 7/group for LPS and LPS + OLDA groups, and *n* = 3/group for saline controls). *P < 0.05, **P < 0.01, ***P < 0.001, two-tailed Mann–Whitney *U* test

To investigate the effects of exogenous OLDA on TRPV1-dependent modulation of systemic inflammation, we challenged wild-type and *Trpv1*^*−/−*^ mice intravenously (i.v.) with the TLR4 agonist LPS, and immediately thereafter treated them i.v. with OLDA or vehicle. Treatment with OLDA, despite a short half-life of roughly 90 s (Additional file 1: Fig. S1A), led to marked attenuation of the early pro-inflammatory response in the endotoxemic wild-type mice (Fig. 1A). This was characterized by reductions in multiple pro-inflammatory cytokines and chemokines, including IL-6 (*P* < 0.0001), TNF-α (*P* < 0.0001), IL-1β (*P* < 0.0001), IL-12p70 (*P* = 0.0001), and pro-inflammatory chemokines CCL2 (*P* < 0.0001), CCL3 (*P* = 0.003), CCL5 (*P* < 0.0001), and CXCL1 (*P* = 0.02) in the plasmas of endotoxemic mice treated with OLDA versus vehicle (Table 1, Fig. 1A). We also observed a striking increase in plasma levels of the anti-inflammatory cytokine IL-10 in OLDA-treated mice (*P* < 0.0001; Table 1, Fig. 1A). The IL-6/IL-10 ratio is often reported as an integrative marker of the pro-inflammatory balance, with higher levels associated with a worse outcome of critical illnesses [48, 49]. Compared with the vehicle, treatment with OLDA led to a roughly tenfold decrease in the plasma IL-6/IL-10 ratio (Fig. 1A; *P* < 0.0001). Furthermore, post-treatment with OLDA at *T* = 2 h after LPS challenge, once inflammation was established, led to increased IL-10 levels in plasmas collected at *T* = 4 h after OLDA (Additional file 1: Fig. S1B). Post-treatment with OLDA did not decrease IL-6 or CCL-2 (data not shown). OLDA upregulated IL-10 and reduced multiple pro-inflammatory cytokines in wild-type, but not *Trpv1*^*−/−*^ endotoxemic mice (Fig. 1A and Additional file 1: Fig. S1C). Similar to our results with LPS treatment, in mice challenged i.v. with the TLR1/2 agonist, tripalmitoyl-S-glyceryl cysteine (Pam3Cys), OLDA administration reduced plasma levels IL-6 and CCL2, and upregulated IL-10 in Pam3Cys-treated wild-type, but not *Trpv1*^*−/−*^ mice. Thus OLDA exerts TRPV1-dependent anti-inflammatory effects in TLR2 (Pam3Cys) and TLR4 (LPS)-induced inflammation (Additional file 1: Fig. S1D). We determined the effects of OLDA on mouse sepsis scores (MSS) which reflect the overall severity of illness and are predictive of mortality in mouse sepsis models [43]. Mice treated with LPS alone developed signs of illness that peaked in severity approximately 12 h after LPS administration and were sustained through the 24-h endpoint. Mouse sepsis score were decreased in OLDA-treated mice at 12 h (*P* = 0.008) and 24 h (*P* = 0.02) after LPS administration (Fig. 1B1–B2). In time-course experiments using LPS-treated wild-type mice, the administration of OLDA administration induced an early peak of IL-10 at 2 h (*P* < 0.001), and at 12- and 24-h IL-10 levels were comparable between endotoxemic mice treated with OLDA and vehicle (Fig. 1C). In contrast, pro-inflammatory mediators including IL-6 and CCL-2 were sustainably reduced across 24 h (data not shown). We used flow cytometry to assess the immune response in the blood, bone marrow, and spleen at *T* = 2, 12, and 24 h in endotoxemic mice treated with OLDA or vehicle. There were no differences among leukocyte subsets in the blood or bone marrow between mice treated with OLDA *versus* vehicle (Additional file 1: Fig. S2A-C). No differences were observed between the splenic Tregs (Foxp3+CD25+CD4+) subset in mice treated with OLDA versus vehicle to explain the upregulation of IL-10 (Additional file 1: Fig. S3A). In contrast, the expression of activation markers by subsets of splenic immune cells were substantially reduced in OLDA-treated mice. Spleen granulocytes expressed lower levels of CD40 and MHC2 that was maintained up to 24 h in OLDA-treated mice (Additional file 1: Fig. S3B). The expression of CD69 was reduced on CD4+ T cells and NK cells, and CD25 expression was reduced on B cells and NK cells (Fig. 1D, Additional file 1: Fig. S3C). This immunomodulatory effect on the innate and the adaptive response, points to a non-cell-specific action of OLDA, and we speculate results from the immunosuppressive effects of IL-10.

**Table 1:** Exogenous OLDA administration modulates circulating cytokines and chemokines during acute inflammation

Inflammatory mediator (mean ± SD)	LPS + vehicle	LPS + OLDA	*P* (Mann–Whitney)
IL-1β (pg/ml)	101 ± 44	40 ± 29	0.0005^χ^
IL-2 (pg/ml)	9.3 ± 4.7	6.6 ± 2	0.04^χ^
IL-4 (pg/ml)	1.9 ± 0.24	1.7 ± 0.28	0.06
IL-5 (pg/ml)	29 ± 6.1	15 ± 8.4	0.0003^χ^
IL-6 (ng/ml)	927 ± 26	477 ± 196	< 0.0001^χ^
IL-9 (pg/ml)	165 ± 26	60 ± 28	< 0.0001^χ^
IL-10 (ng/ml)	2.1 ± 0.5	13 ± 7.9	< 0.0001^θ^
IL-12p70 (pg/ml)	26 ± 4.7	19 ± 1.8	0.0001^χ^
IL-13 (pg/ml)	4.7 ± 1.1	3.6 ± 0.32	0.003 ^χ^
IL-17A (pg/ml)	4.4 ± 2	3.4 ± 1.2	0.26
IL-18 (ng/ml)	2.4 ± 0.17	2.2 ± 0.15	0.06
IL-22 (ng/ml)	19 ± 7	2.8 ± 2.3	< 0.0001^χ^
IL-23 (pg/ml)	21 ± 16	15 ± 17	0.0158^χ^
IL-27 (pg/ml)	100 ± 29	41 ± 22	< 0.0001^χ^
GM-CSF (pg/ml)	22 ± 2.9	15 ± 1.8	< 0.0001^χ^
TNF-α (ng/ml)	1.1 ± 0.33	0.61 ± 0.13	< 0.0001^χ^
IFN-γ (pg/ml)	13 ± 4.7	8.6 ± 1.7	0.01^χ^
MCP-1/CCL-2 (ng/ml)	91.3 ± 22	55.7 ± 19	< 0.0001^χ^
MIP-1α/CCL-3 (ng/ml)	1.2 ± 0.2	0.75 ± 0.2	< 0.0001^χ^
MIP-1β/CCL-4 (ng/ml)	5.8 ± 1.3	5.1 ± 2	0.52
RANTES/CCL-5 (ng/ml)	0.8 ± 0.17	0.4 ± 0.15	< 0.0001^χ^
MCP-3/CCL-7 (ng/ml)	1.2 ± 0.16	0.99 ± 0.1	0.0028^χ^
Eotaxin/CCL-11 (ng/ml)	1 ± 0.12	0.81 ± 0.09	< 0.0001^χ^
Gro-α/CXCL-1 (ng/ml)	12 ± 5.7	6.1 ± 4.3	0.016^χ^
IP-10/CXCL-10 (pg/ml)	476 ± 37	359 ± 75	< 0.0001^χ^

Wild-type mice were treated with LPS (1 mg/kg, i.v.) and immediately thereafter with OLDA (10 mg/kg, i.v.) or vehicle (i.v.). Cytokines and chemokines were quantified in plasma at *T* = 2 h using Procartaplex™ immunoassay technology (Thermo Fisher Scientific) (9- to 12-week male and female, *n* = 11–12/group)

^θ^Indicates significant upregulation

^χ^Indicates significant downregulation

### OLDA administration reduces the severity of LPS-induced acute lung injury (ALI)

**Fig. 2:**
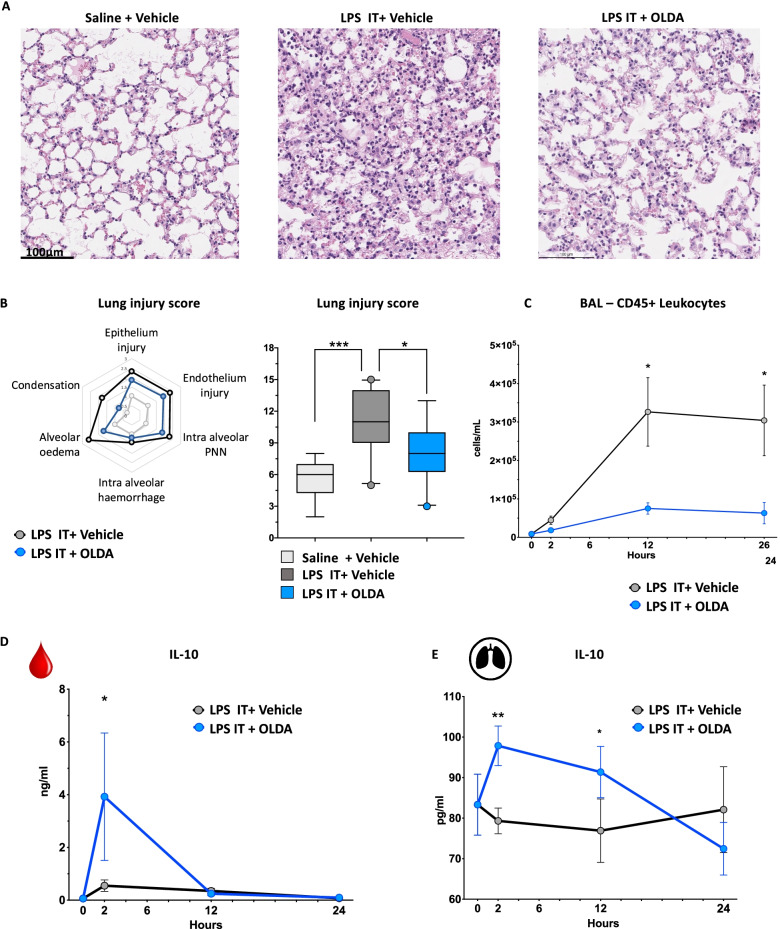
OLDA administration ameliorates LPS-induced acute lung injury. Wild-type mice were challenged with LPS (5 mg/kg, i.t.) and immediately thereafter were treated with OLDA (5 mg/kg, i.v.) or vehicle (i.v.). **A** Representative H&E-stained sections of lungs (original magnification ×20) collected from mice 24 h after challenge with saline, LPS i.t. + vehicle, or LPS i.t. + OLDA. **B** Lung injury scores were assessed by analysis of epithelial and endothelial injury, alveolar hemorrhage, alveolar edema, PMN infiltration, and condensation. **C** Mice treated with OLDA had reduced leucocyte counts (CD45+ cells) in BAL fluid 24 h after induction of acute lung injury. **D** OLDA treatment induced IL-10 upregulation in the plasma at 2 h. **E** OLDA treatment induced IL-10 upregulation in BALF at 2 and 12 h (12-week-old male, *n* = 5 per group and per time point for all panels). *P < 0.05, **P < 0.01, ***P < 0.001, LPS-treated mice vehicle vs. OLDA, two-tailed Mann–Whitney *U* test at each time point

The effects of OLDA were studied in mice with ALI induced by intratracheal (i.t.) administration of LPS. As compared with vehicle, treatment with OLDA reduced the severity of histological lesions 24 h after LPS challenge, and it alleviated alveolar edema, lung condensation, and intra-alveolar polymorphonuclear counts, as shown in representative images (Fig. 2A), and in significantly reduced lung injury scores (Fig. 2B; *P* = 0.04). Based on flow cytometry, OLDA reduced the number of CD45+ leukocytes in the bronchoalveolar lavage (BAL) fluid 12 and 24 h after i.t. LPS challenge (Fig. 2C). Finally, IL-10 levels were increased in the plasma 2 h, and in the BAL fluid 2 and 12 h after i.t. LPS challenge (Fig. 2D, E). Thus, we observed that OLDA dampens the systemic and lung pro-inflammatory response and alleviates organ injury.

### OLDA reduces inflammation and the severity of *Staphylococcus aureus *(*S. aureus*)-induced pneumopathy without affecting antibacterial defenses

**Fig. 3:**
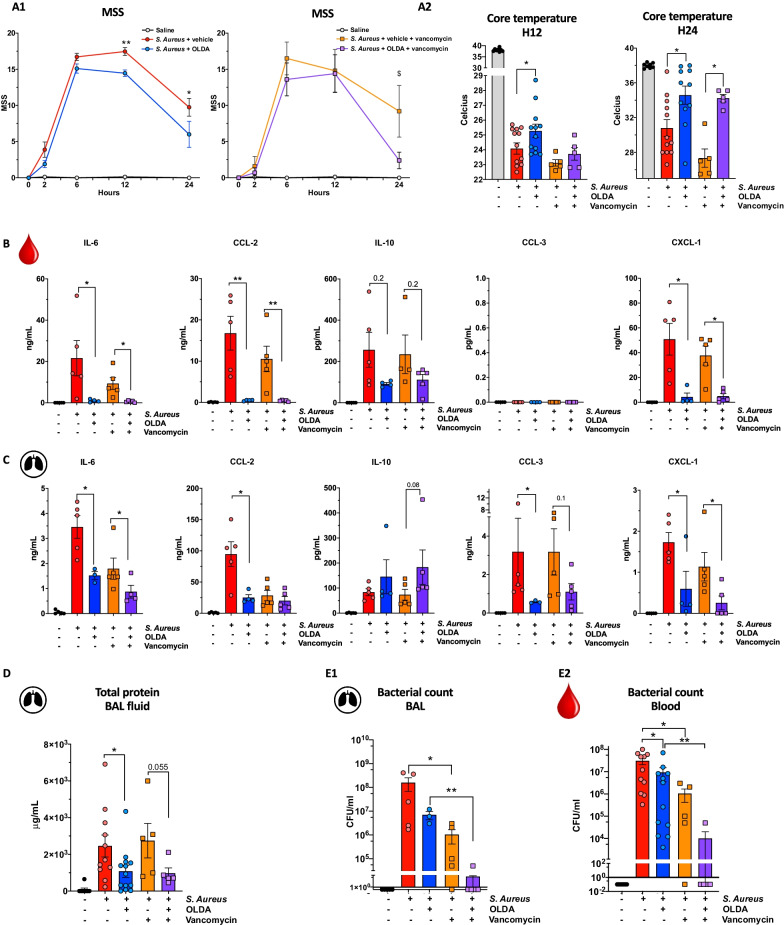
OLDA administration improves outcomes of antibiotic-treated or untreated *S. aureus*-induced pneumopathy. Wild-type mice were treated with *S. aureus* (MSSA, 1 × 10^7^ CFU/25 g body weight, i.t.) and immediately thereafter with OLDA (10 mg/kg, i.v.) or vehicle (i.v.). They received a second dose of OLDA (10 mg/kg) or vehicle at 2 h. Additionally, mice were randomly assigned to receive vancomycin (10 mg/kg, i.p.) or carrier (i.p.) at 6 and 12 h. **A1** Treatment with OLDA reduced MSS at 12 and 24 h in mice that did not receive vancomycin, and at 24 h in vancomycin-treated mice. **A2** Treatment with OLDA reduced sepsis-induced hypothermia severity in vancomycin-treated and non-treated mice (10- to 12-week male, *n* = 12/group for mice that did not receive vancomycin, *n* = 5/group for mice that did not receive vancomycin). **B**, **C** Treatment with OLDA reduced **B** plasma levels of IL-6, CCL-2, CXCL-1, and **C** BAL fluid levels of IL-6, CCL2, CCL3, CXCL1 at *T* = 24 h after induction of *S. aureus* pneumonia (12-week male, *n* = 5/group). **D** Shows that OLDA treatment reduced lung capillary leak assessed by total protein in BAL fluid at 24 h in both mice treated with or without vancomycin (10- to 12-week male, *n* = 12/group for mice that did not receive vancomycin, *n* = 5/group for mice that did not receive vancomycin). **E** 24 h after inducing *S. aureus* pneumonia, CFU counts were not significantly in BAL fluid (*n* = 3–5/group), and blood (10- to 12-week male, *n* = 12/group for mice that did not receive vancomycin, *n* = 5/group for mice that did not receive vancomycin) of mice that did or did not receive OLDA, suggesting that OLDA does not impair blood bacterial clearance (10- to 12-week male, *n* = 42). *P < 0.05, **P < 0.01, ***P < 0.001, LPS-treated mice vehicle vs. OLDA, two-tailed Mann–Whitney *U* test at each time point

IL-10 has been reported to impair macrophage antimicrobial defenses and reduce activation of adaptive immune cells, potentially leading to increased susceptibility to infections [50–53]. We investigated the effect of OLDA on inflammatory and clinical outcomes in mice with *S. aureus* pneumonia. Two doses of OLDA were administered to each mouse, one immediately after i.t. challenge with *S. aureus*, and the second at *T* = 2 h. Vancomycin or saline was administered at *T* = 6 and 12 h. Treatment with OLDA reduced clinical signs of sepsis severity, including mouse sepsis scores (Fig. 3A1) and the degree of hypothermia (Fig. 3A2) in mice treated with and without vancomycin. Treatment with OLDA reduced plasma levels of IL-6 (*P* = 0.01), CCL2 (*P* = 0.02), and CXCL1 (*P* = 0.02) at 24 h (Fig. 3B), and reduced BAL fluid levels of IL-6 (*P* = 0.008) and CXCL1 (*P* = 0.04) in mice that did and did not receive vancomycin (Fig. 3C). OLDA treatment was associated with reduced BAL fluid levels of CCL2 (*P* = 0.03) and CCL3 (*P* = 0.04) only in mice that did not receive vancomycin (Fig. 3C). Treatment with vancomycin reduced lung injury scores in all mice with *S. aureus* pneumonia (*P* = 0.03), but there were no differences in lung injury scores between mice treated with OLDA and vehicle (Additional file 1: Fig. S4A). Notably, OLDA treatment reduced total protein concentration levels in the BAL fluid of vancomycin-treated and untreated groups (Fig. 3D). This suggests that OLDA reduces lung capillary leakage, which is a prominent pathophysiological feature of ALI. OLDA also reduced mobilization of Ly6C hi, and Ly6C low monocyte recruitment towards the infected lung (Additional file 1: Fig. S4B). Regardless of antibiotic therapy, OLDA blunted the activation of spleen T cells, reduced proliferation and activation of CD4 effector (CD4+CD62LnegCD44lo) T cells (reduced CD25 and CD69) and spleen monocytes (reduced MHC2), and decreased the mobilization of spleen Ly6C hi monocytes (Additional file 1: Fig. S5A, B).

Notably, at 24 h post-infection, mice treated with OLDA and vehicle had comparable bacterial counts (CFU) in their BAL fluid and blood (Fig. 3E1, E2). These findings indicate that despite potent anti-inflammatory effects, OLDA did not impair early bacterial clearance from the lungs or bloodstream.

### Neuronal TRPV1 mediates the upregulation of systemic IL-10 by OLDA in endotoxemic mice

**Fig. 4:**
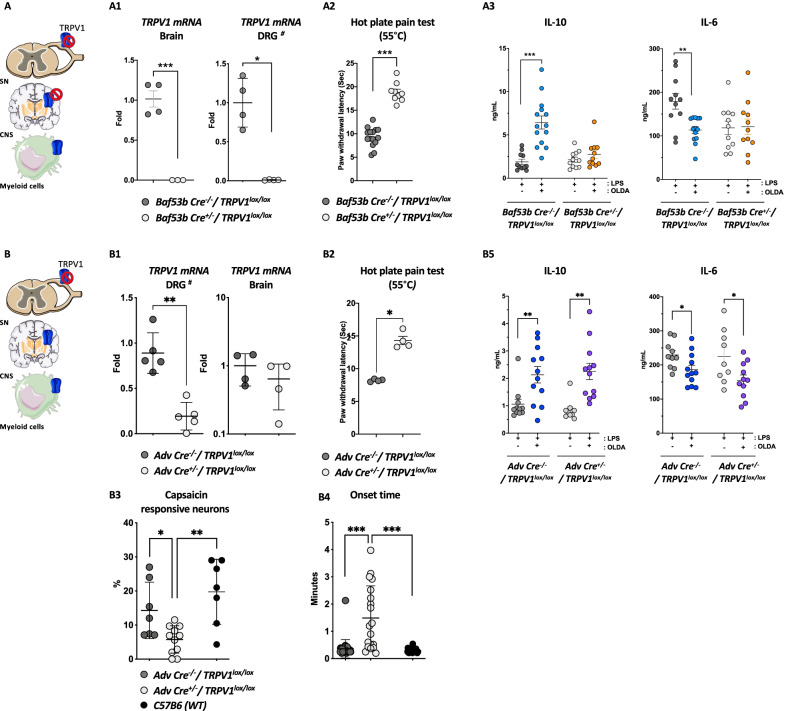
OLDA upregulates circulating IL-10 in endotoxemic mice via TRPV1-expressing neurons of the central nervous system. **A** OLDA induces IL-10 via neuronal TRPV1: **A1** *Baf53b-Cre*^+/−^*/TRPV1*^*lox/lox*^ mice had reduced brain and DRG TRPV1 gene expression. ^***#***^For DRG qPCR, each dot represents a pool from 1–2 animals. **A2** *Baf53b-Cre*^±^*/ TRPV1*^*lox/lox*^ mice had attenuated responses to heat-sensitivity testing (8- to 12-week female mice). **A3** Pan-neuronal TRPV1 knockdown in *Baf53b-Cre*^+/−^*/TRPV1*^*lox/lox*^ mice fully abrogated the upregulation of IL-10 and the downregulation of IL-6 induced by OLDA (10 mg/kg, i.v.) in mice treated with LPS (1 mg/kg, i.v.) (12-week male, *n* = 11–14/group). **B** OLDA does not act via PNS TRPV1; **B1** *Adv Cre*^+/−^*/TRPV1*^*lox/lox*^ mice had reduced TRPV1 expression in their DRG but not their brains. ^***#***^For DRG qPCR, each dot represents a pool from 1–2 animals. **B2** *Adv Cre*^+/−^*/TRPV1*^*lox/lox*^ mice displayed attenuated responses to heat-sensitivity testing (8- to 12-week female mice). **B3**–**B4** Calcium imaging shows a decreased percentage of capsaicin-responsive neurons in DRG neurons *Adv Cre*^+/−^*/TRPV1*^*lox/lox*^ compared to *Adv Cre*^*−/−*^*/TRPV1*^*lox/lox*^ and wild-type mice, and longer onset response time (*n* = 3 mice/group; number of neurons imaged = 19–31 neurons/genotype). **B5** Peripheral neuron TRPV1 knockdown in *Adv Cre*^+/−^*/TRPV1*^*lox/lox*^ mice did not abrogate the IL-10 upregulation or IL-6 downregulation induced by OLDA (10 mg/kg, i.v.) in mice treated with LPS (1 mg/kg, i.v.) (10- to 12-week male and female, *n* = 10–12/group). For all panels, *P < 0.05, **P < 0.01, ***P < 0.001, two-tailed Mann–Whitney *U* test

We hypothesized that OLDA upregulates systemic IL-10 production via neuronal TRPV1 based on our prior finding that NADA induced IL-10 via non-hematopoietic TRPV1 [37], the known role of neuronal TRPV1 in inflammatory pain, and the high level of TRPV1 expression by neurons [54]. We determined the role of neuronal TRPV1 in the anti-inflammatory actions of OLDA in endotoxemia by comparing responses of mice with pan-neuronal TRPV1 knockdown that were produced by breeding *BAF53b-Cre*^+/−^ mice [55], with *Trpv1*^*lox/lox*^ mice to generate *BAF53b-Cre*^+/−^*/Trpv1*^*lox/lox*^ mice with control *BAF53b-Cre*^*−/−*^*/Trpv1*^*lox/lox*^ mice with normal TRPV1 expression. We used qPCR to verify TRPV1 gene knockdown in the brain (*P* < 0.001) and in DRG (Fig. 4A1; *P* = 0.02), and lack of TRPV1 knockdown in other organs (data not shown). We confirmed knockdown of neuronal TRPV1 at the functional level by demonstrating that *BAF53b-Cre*^±^*/Trpv1*^*lox/lox*^ mice have attenuated responses to the hot-plate testing compared to *BAF53b-Cre*^*−/−*^*/Trpv1*^*lox/lox*^ (control) mice (Fig. 4A2; *P* < 0.001). Treatment with OLDA led to increased IL-10 (*P* < 0.001) and decreased IL-6 (*P* = 0.009) in endotoxemic control (*BAF53b-Cre*^*−/−*^*/Trpv1*^*lox/lox*^) mice. Remarkably, OLDA did not upregulate IL-10, and did not downregulate IL-6 in pan-neuronal TRPV1 knockdown (*BAF53b-Cre*^+/−^*/Trpv1*^*lox/lox*^) (Fig. 4A2, Additional file 1: Fig. S6A). These data indicate that OLDA exerts its anti-inflammatory effects via neuronal TRPV1.

### TRPV1 expressed by peripheral nervous system neurons does not mediate the upregulation of systemic IL-10 by OLDA in endotoxemic mice

TRPV1 is highly expressed by neurons in the PNS [10, 56–60]. Therefore, we assessed the role of sensory neuron TRPV1 in the anti-inflammatory effects of OLDA. We bred *Advillin-Cre*^+/−^ mice [61] with *Trpv1*^*lox/lox*^ mice to generate *Advillin-Cre*^+/−^/*Trpv1*^*lox/lox*^ mice with TRPV1 knockdown in their PNS neurons (sensory neurons of the DRG, and the sympathetic, parasympathetic, and enteric nervous systems). We used qPCR to verify that *Advillin-Cre*^+/−^/*Trpv1*^*lox/lox*^ mice had TRPV1 knockdown in their DRG but not in their brains, and that control (*Advillin-Cre*^−/−^/*Trpv1*^*lox/lox*^) mice had preserved DRG and brain TRPV1 expression (Fig. 4B1). We further confirmed sensory neuron TRPV1 knockdown in *Advillin-Cre*^+/−^/*Trpv1*^*lox/lox*^ mice by demonstrating that they have reduced responses to heat compared with control (A*dvillin-Cre*^−/−^/*Trpv1*^*lox/lox*^) mice (Fig. 4B2; *P* = 0.02). We corroborated knockdown of TRPV1 in DRG neurons, using calcium imaging to measuring capsaicin-induced calcium responses of DRG neurons [46]. The percentage capsaicin-responsive neurons was decreased, and the average onset times of capsaicin-evoked responses were longer in DRG neurons from *Advillin-Cre*^+/−^*/Trpv1*^*lox/lox*^ as compared with *Advillin-Cre*^*−/−*^*/Trpv1*^*lox/lox*^ or wild-type mice (Fig. 4B4–6; *P* < 0.05). In contrast to our results using mice with pan-neuronal TRPV1 knockdown (Fig. 4A3), the anti-inflammatory effects of OLDA were fully preserved in *Advillin-Cre*^+/−^*/Trpv1*^*lox/lox*^ mice (Fig. 4B3 and Additional file 1 Fig. S6B). These results indicate that PNS TRPV1 neurons do not mediate the TRPV1-dependent anti-inflammatory effects of OLDA.

### Circulating monocytes/macrophages mediate the upregulation of systemic IL-10 by OLDA in endotoxemic mice

**Fig. 5:**
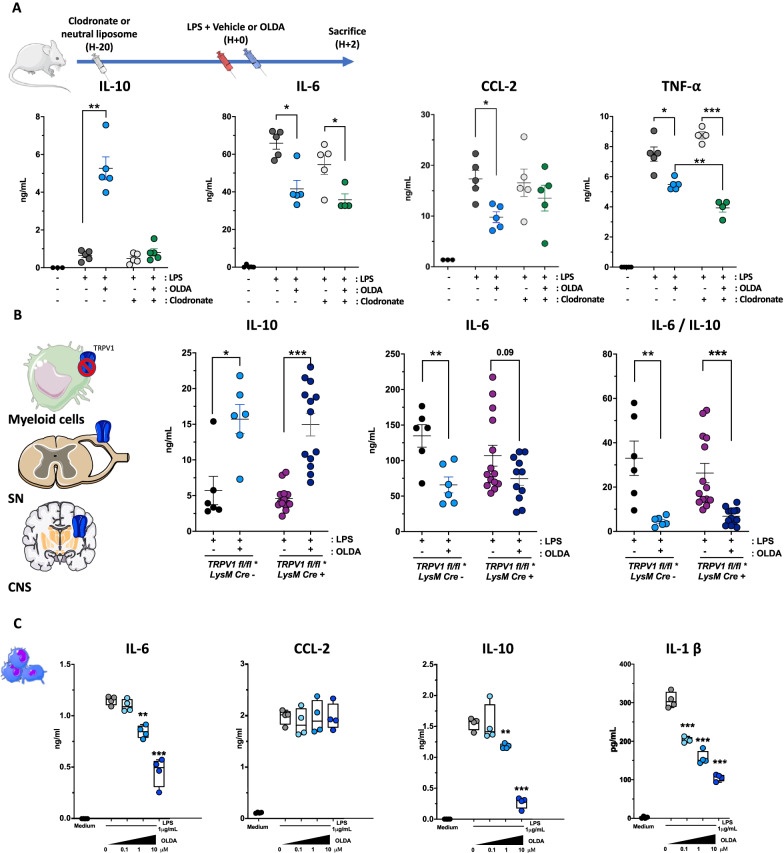
OLDA-induced upregulation of IL-10 in endotoxemic mice depends on circulating monocytes/macrophages. **A** OLDA-induced IL-10 production is dependent on circulating monocytes/macrophages in endotoxemic mice: Clodronate-mediated depletion of phagocytic myeloid cells reversed OLDA-induced IL-10 upregulation, and abrogated, but did not fully reverse, the downregulation of pro-inflammatory cytokines (IL-6, CCL-2, TNF-α) in mice treated with LPS (1 mg/kg, i.v.; 10- to 12-week male, *n* = 5/group for all treatment conditions; *n* = 3 mice for untreated controls). **B** Myeloid specific TRPV1 deficiency (*LysM Cre*^+/−^*/TRPV1*^*lox/lox*^) did not abrogate the anti-inflammatory effects of OLDA in mice treated with LPS (1 mg/kg, i.v.; 10- to 12-week male and female, *n* = 6–12/group). **C** Ex vivo*,* OLDA directly reduced secretion of pro-inflammatory mediators (IL-6, IL-1β) and IL-10 by LPS-stimulated bone marrow-derived macrophages (cells differentiated from 10-week wild-type male, *n* = 4). *P < 0.05, **P < 0.01, ***P < 0.001, LPS + vehicle compared with LPS + OLDA, two-tailed Mann–Whitney *U* test

Clodronate-depletion experiments were performed to define the role of monocytes/macrophages in the upregulation of IL-10 by OLDA. Mice received clodronate liposome or neutral liposomes (controls) 20 h prior to the LPS challenge. The depletion of monocytes/macrophages entirely reversed the OLDA-induced IL-10 upregulation, suggesting that monocytes/macrophages are required for the upregulation of IL-10 (Fig. 5A). However, OLDA still partially reduced IL-6, CCL-2, and TNF-α plasma levels in clodronate-treated mice (Fig. 5A). This finding suggests first that the downregulation of pro-inflammatory cytokines by OLDA is not solely dependent on the upregulation of IL-10, and second, that OLDA may also exert direct anti-inflammatory effect on immune cell subsets other than those of the myeloid mononuclear compartment.

### Myeloid TRPV1 does not mediate the anti-inflammatory actions of OLDA

We assessed the role of TRPV1 expressed by leukocytes on the anti-inflammatory properties of OLDA using transgenic mice that lack TRPV1 expression in the myeloid compartment, that were generated by breeding *LysM-Cre*^+/−^ mice with *Trpv1*^*lox/lox*^ mice to produce *LysM-Cre*^+/−^*/Trpv1*^*lox/lox*^ mice, which have myeloid TRPV1 knockdown, and *LysM-Cre*^*−/−*^*/Trpv1*^*lox/lox*^ mice, which express TRPV1 in myeloid cells. We verified knockdown of TRPV1 expression in myeloid cells but not in neurons of *LysM-Cre*^+/−^*/Trpv1*^*lox/lox*^ mice using qPCR, and further confirmed lack of neuronal TRPV1 knockdown by demonstrating their retained thermosensitivity using hot-plate testing (data not shown). In both *LysM-Cre*^+/−^*/Trpv1*^*lox/lox*^ and *LysM-Cre*^*−/−*^*/Trpv1*^*lox/lox*^ mice, treatment with OLDA led to comparable degrees of upregulation of IL-10 (*P* = 0.05 and *P* < 0.001, respectively, OLDA versus vehicle; Fig. 5B). There was only a trend towards a decrease in the reduction of IL-6 in OLDA *versus* vehicle-treated *LysM-Cre*^+/−^*/Trpv1*^*lox/lox*^ mice (*P* = 0.09). Nevertheless, the IL-6/IL-10 ratio revealed that OLDA-treated *LysM-Cre*^+/−^*/Trpv1*^*lox/lox*^ mice have a similar reduced inflammatory response compared to *LysM-Cre*^*−/−*^*/Trpv1*^*lox/lox*^ (Fig. 5B). These data indicate that the IL-10 inducing actions of OLDA in acute inflammation are not directly mediated by TRPV1 expressed by myeloid cells, and thus further support a role for neuronal TRPV1 in OLDA’s anti-inflammatory effects.

### OLDA reduces inflammatory activation of bone marrow-derived macrophages (BMDM), astrocytes, and endothelial cells in vitro

We assessed the effects of OLDA in vitro on LPS-induced activation of mouse BMDM, human astrocytes and human lung microvascular endothelial cells. Similar to our results in endotoxemic mice, OLDA reduced LPS-induced secretion of pro-inflammatory cytokines (e.g., IL-6, IL-1β) by BMDM (Fig. 5C; *P* < 0.01). In contrast to the IL-10 inducing properties of OLDA in mice, in vitro treatment with OLDA led to reduced LPS-induced IL-10 production by BMDM (Fig. 5C, *P* = 0.002). Also, co-treatment with OLDA and IL-10 additively reduced production of pro-inflammatory cytokines by LPS-stimulated BMDM (Additional file 1: Fig. S7A). OLDA also reduced LPS-induced production of IL-6 (*P* = 0.01), CCL2 (*P* = 0.009), and IL-8 (*P* = 0.004) by human lung microvascular endothelial cells (Additional file 1: Fig. S7B). This is of interest as microvascular endothelial cells are a major source of cytokines and chemokines during sepsis and other inflammatory critical illnesses [62]. Finally, OLDA reduced LPS-induced IL-6 (*P* < 0.001) and CCL2 (*P* < 0.001), but not IL-8, by human astrocytes (Additional file 1: Fig. S7C). Thus, direct treatment with OLDA is able to reduce pro-inflammatory cytokine and chemokine production but does not upregulate IL-10 production by multiple cell subsets (BMDM, endothelial cells, astrocytes). The lack of upregulation of IL-10 by cultured cell populations supports the concept that a multi-cellular network is responsible for the upregulation of IL-10 in OLDA-treated endotoxemic mice.

### Acute inflammation alters brain and plasma levels of endogenous lipids, including endovanilloids and endocannabinoids

**Fig. 6:**
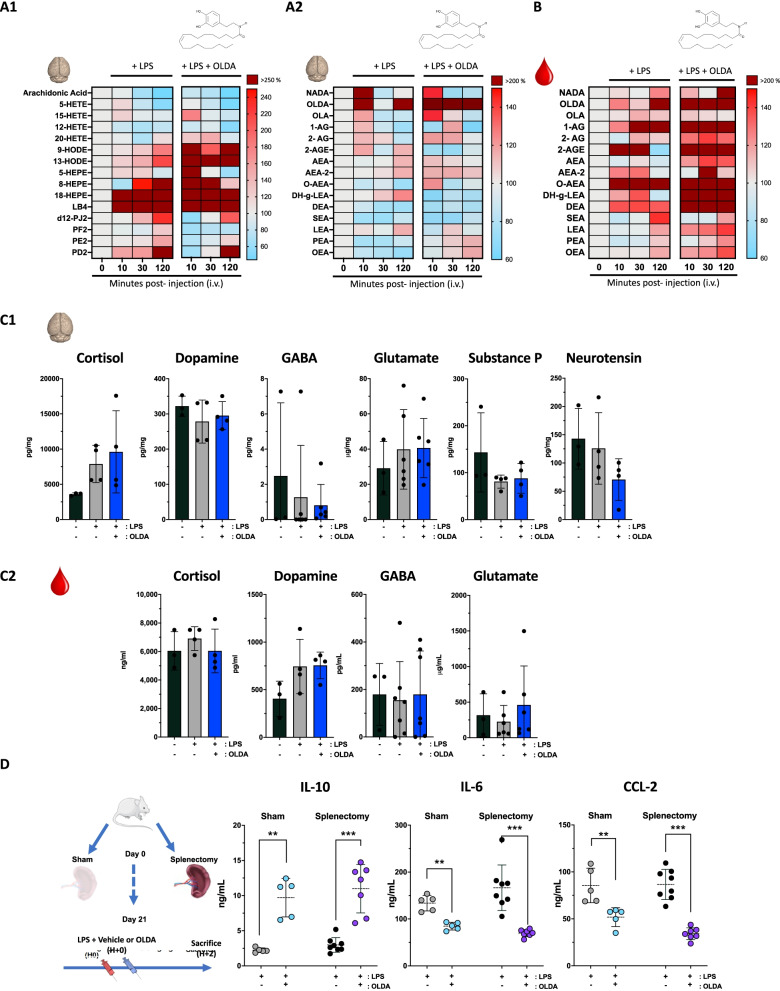
Endogenous neurolipid modulation during acute inflammation and the effects of exogenous OLDA on neuroinflammatory mediators. **A** Regulation of endogenous neurolipids, including arachidonic acid and its metabolites (prostaglandin, leukotriene B4, eicosapentaenoic acid, hydroxyeicosatetraenoic acid and octadecadienoic acid) and conjugation products (*N*-acyl dopamines, acyl-glycerol and *N*-acylethanolamines) following LPS ± exogenous vanilloid (OLDA) injection. LPS injection provoked acute changes in *N*-acyl-dopamines and acyl-ethanolamines in brain and plasma: Wild-type mice were challenged with LPS (3 mg/kg, i.v.) ± OLDA (10 mg/kg, i.v.). Brain and plasma were harvested at 10, 30 and 120 min (12-week male, *n* = 4–8 mice per time point, respectively, at 0, 10, 30 and 120 min after LPS injection). **A1** In the brain, arachidonic acid and its hydroxyeicosatetraenoic acid (HETE) metabolites were significantly downregulated during acute inflammation. **A2**, **B** Conversely, arachidonic acid metabolites from the prostaglandin (PD2, PE2 and D12PJ2) leukotriene B4, eicosapentaenoic and octadecadienoic acid families were upregulated. OLDA was barely detectable at baseline, in brain and plasma and trended towards increased in blood and plasma during the first 120 min following LPS injection, as did acyl-glycerol (1-AG, 2-AGE) and *N*-acylethanolamines in the plasma. Results are shown as a heat map, baseline levels being considered as reference (100%) for each metabolite (12-week male, *n* = 4–8 mice per time point, respectively, at 0, 10, 30 and 120 min after LPS injection). **C** Changes in levels of hormones or peptide neuromodulators in **C1** total brain extracts and **C2** plasmas from wild-type mice, at baseline and 2 h after i.v. challenge with LPS (1 mg/kg) followed by either OLDA (10 mg/kg) or carrier (9-week male, *n* = 4/group for cortisol, dopamine, substance P and neurotensin; 10-week-old male, 13-week female, *n* = 7–10/group for GABA; 8- to 9-week female, *n* = 6–9/group for glutamate). **D** The vanilloid neuro-immune anti-inflammatory reflex does not require the spleen: Splenectomy performed 3 weeks prior to induction of endotoxemia and treatment with OLDA did not abrogate the anti-inflammatory effects of OLDA. These results suggest that the neuro-immune action of OLDA is independent of the vagal–splenic anti-inflammatory pathway, and that bone marrow rather than spleen Mo-MDSCs produce IL-10 in response to co-treatment with LPS + OLDA. Plasma cytokines were quantified in plasma at *T* = 2 h after LPS challenge (1 mg/kg i.v.) and OLDA (10 mg/kg i.v.) or carrier (i.v.) (12- to 13-week male, *n* = 5–8/group). **P < 0.01, ***P < 0.001, LPS-treated mice vehicle vs. OLDA, two-tailed Mann–Whitney *U* test

To test the hypothesis that acute inflammation modulates endovanilloid and endocannabinoid mediators, we quantified levels of arachidonic acid, its metabolites (prostaglandins, hydroxyeicosatetraenoic acid (HETE), eicosapentaenoic acid (HEPE), octadecadienoic acid (HODE)), and vanilloid/cannabinoid conjugation products (*N*-acyl dopamines, *N*-acylethanolamines, 2-acyl glycerols) in wild-type mice 10 min, 30 min, and 120 min after challenge with LPS (3 mg/kg, i.v.). Arachidonic acid concentrations in the brain were reduced following LPS injection (1525 ± 560 pg/mg baseline versus 684 ± 559 pg/mg at 120 mn, *P* = 0.03) (Fig. 6A1). Conversely, arachidonic acid metabolites from the prostaglandin family (PD2, PE2 and D12PJ2) were increased at 120 min as compared to baseline (respectively, *P* = 0.008, *P* = 0.002 and *P* = 0.001), as were as leukotriene B4, eicosapentaenoic and octadecadienoic acids (Fig. 6A1). These results suggest the activation of both the cyclooxygenase and lipoxygenase pathways in the CNS following inflammatory stimulus. OLDA was barely detectable in the brain at baseline (0.51 ± 0.18 pg/mg) and trended towards an increase at 10 min and 120 min (Fig. 6A2; 2.2 ± 3.2 pg/mg and 1.6 ± 0.99 pg/mg, respectively; *P* = 0.08, and *P* = 0.16) after LPS challenge. NADA was also barely detectable in the brain at baseline (0.11 ± 0.08), and there was a non-significant trend towards increased NADA in the brain at 10 min (0.25 ± 0.2 pg/mg; *P* = 0.07), but not at later time points (Fig. 6B). Plasma levels of OLDA were increased from baseline (57.5 ± 33 pg/ml), at 120 min after LPS challenge (Fig. 6B; 1119 ± 1808 pg/ml; *P* = 0.08). Similar trends were obtained with some of the other endocannabinoids and endovanilloids, including 1-arachidonoyl glycerol (1-AG) (+ 197% at 30 mn, P = 0.07), *O*-arachidonoyl ethanolamine (O-AEA) (+ 233% at 30 mn, *P* = 0.15) and *N*-docosatetraenoylethanolamide (DEA) (+ 138%% at 30 mn, *P* = 0.09) (Fig. 6B). These results suggest that acute systemic inflammation modulates systemic and brain production of endogenous neurolipids belonging to the extended endovanilloid and endocannabinoid systems.

### Administration of exogenous OLDA modulates levels of other endovanilloid and endocannabinoids in vivo in endotoxemic mice

We next assessed the effects of i.v. administration of exogenous OLDA on levels of other endovanilloids and endocannabinoids in endotoxemic mice. Despite the short half-life (90 s, Additional file 1: Fig. S1A) of OLDA in the bloodstream based on pharmacokinetic studies, OLDA levels were substantially increased in the brains at 10 min (1856 ± 770 pg/ml, *P* = 0.005 versus baseline) and 30 min (451 ± 110 pg/ml, *P* = 0.02 versus baseline) (Fig. 6A2). Plasma levels of OLDA were also increased at 10 (489 ± 38 ng/ml, *P* = 0.005 versus baseline) and at 30 min (118 ± 17 ng/ml, *P* = 0.03 versus baseline) in endotoxemic mice treated with OLDA (Fig. 6B). These results suggests that exogenously administered OLDA can cross the blood–brain barrier and supports the concept that exogenous OLDA exerts its anti-inflammatory actions via activity at CNS TRPV1. Additionally, the i.v. administration of OLDA upregulated brain and plasma levels of the 2-acylglycerols and N-acylethanolamines within 2 h following the injection (Fig. 6A1–B).

### OLDA treatment does not modulate plasma or brain levels of cortisol, neurotransmitters, or substance P

TRPV1 is involved in neuronal function [63, 64], and our studies indicate that OLDA and NADA have potent TRPV1-dependent immunomodulatory properties. To investigate the mechanistic links connecting the engagement of TRPV1 neurons by OLDA and the upregulation of IL-10 production by circulating monocytes/macrophages, we measured plasma and brain levels of cortisol, several neuromodulators, dopamine, GABA and glutamate. We observed no differences between treated and untreated groups in plasma or brain levels of cortisol, dopamine, GABA, or of Glutamate, or of substance P and neurotensin in the brain (Fig. 6C1), or in the plasma (Fig. 6C2) to parallel the upregulation of IL-10.

### OLDA induces IL-10 in splenectomized endotoxemic mice

Direct innervation of immune tissue such as the bone marrow or the spleen may also modulate the immune response. As yet, the vagal–cholinergic–splenic pathway is the only fully characterized neuro-immune anti-inflammatory reflex [65–68]. However, we observed unequivocally using splenectomized mice, that splenectomy did not abrogate the anti-inflammatory effects of OLDA (Fig. 6D).

## Discussion

Our study identifies a novel neuro-immune anti-inflammatory pathway that is triggered by OLDA activity at TRPV1 neurons of the CNS and leads downstream to upregulated systemic production of IL-10. We found that exogenously administered OLDA has pro-resolving and anti-inflammatory properties and improves clinical outcomes in mice with LPS-induced sterile inflammation and with *S. aureus* pneumonia (Figs. 1, 2, and 3, Additional file 1: Figs. S1, S3–5). Despite having a short half-life and being rapidly cleared from the blood, administration of OLDA to mice with endotoxemia or *S. aureus* pneumonia is associated with immune and functional effects that persist long after blood levels of OLDA are minimal (Figs. 1, 2, and 3, Additional file 1: Fig. S1A). It is unclear whether OLDA is catabolized or distributed into tissues, or if the sustained effects on cytokines and clinical outcomes are secondary to the early upregulation of IL-10. We determined that OLDA TRPV1-dependently upregulates IL-10 and downregulate multiple pro-inflammatory cytokines and chemokines in the blood of mice treated with TLR4 and TLR2 agonists (Fig. 1, Additional file 1: Fig. S1). Our data showing that pan-neuronal TRPV1 knockdown reverses the upregulation of IL-10 and downregulation of IL-6 (Fig. 4A), and that mice with peripheral neuronal TRPV1 knockdown retain their sensitivity to the anti-inflammatory effects of OLDA (Fig. 4B), support the conclusion that TRPV1-expressing neurons of the CNS, not the PNS, mediate OLDA’s anti-inflammatory effects in endotoxemia.

Our finding that depletion of monocytes/macrophages reversed the OLDA-induced upregulation of IL-10 in endotoxemic mice (Fig. 5A) suggests that monocytes/macrophages are essential for upregulation of IL-10. We also found that OLDA directly reduces LPS-induced production of pro-inflammatory cytokines by cultured leukocytes, astrocytes, and endothelial cells, and reduces the secretion of IL-10 by LPS-activated BMDM (Fig. 5C, Additional file 1: Fig. S7). Furthermore, the anti-inflammatory effects of OLDA were retained in mice with myeloid TRPV1 knockdown (Fig. 5B), indicating that OLDA’s anti-inflammatory effects are not mediated by TRPV1 expressed by monocytes/macrophages. These findings are consistent with our prior finding that NADA reduces LPS-induced in vitro activation of leukocytes and endothelial cells via TRPV1-independent mechanisms [37]. Collectively our findings suggest that, under conditions of acute inflammation, OLDA induces IL-10 via a multi-cellular network mediated proximally by CNS TRPV1 neurons and distally by circulating monocytes/macrophages.

TRPV1 is a non-selective cation channel that is well known for its role in inflammatory pain [69–72]. It is highly expressed in PNS neurons, including in the trigeminal nerve and dorsal root ganglia (DRG), and the vagus nerve [10, 56, 70]. TRPV1 expression has also been reported in in the CNS, including in the spinal cord [57] and in the brain [58–60]. Although TRPV1 is expressed in multiple areas of the developing mouse brain, it has more limited expression in the brains of adult mice, restricted to the hippocampus, hypothalamus, and midbrain [58–60]. NADA and OLDA have been detected in the DRG, and in areas of the CNS, including the striatum, hippocampus, cerebellum, thalamus and brainstem [16, 73], which are locations where TRPV1 expression has also been reported [56, 57, 70]. Prior studies on TRPV1 in sepsis have yielded divergent results—some suggesting a protective role, and others a harmful role [74–78]. TRPV1-expressing peripheral sensory neurons have been implicated in the host’s response to pneumonia [79], and enteric infection with *Citrobacter* [80].

Our data are novel in identifying a role for TRPV1 expressed in the CNS, and endovanilloids, such as OLDA and NADA in modulating peripheral immune responses and determining outcomes of acute inflammation and sepsis. The family of TLRs play critical roles in the innate immune response to injury and infection. TLR4 is the host receptor for LPS from Gram-negative bacteria as well as endogenous host factors that are released during injury, such as HMGB1, and TLR2 recognizes microbial lipoproteins, which are expressed by Gram-negative and Gram-positive bacteria. Our finding that OLDA TRPV1-dependently induces an anti-inflammatory cytokine profile in mice treated with TLR2 or TLR4 agonists (Fig. 1; Additional file 1: Fig. S1) suggests that TRPV1 plays a fundamental role in regulating systemic inflammation during systemic infections and injury.

Cytokines and chemokines regulate immune responses and play roles in the pathogenesis of organ failure. Higher levels of pro-inflammatory cytokines or chemokines correlate with worse outcomes in critical illnesses caused by sepsis or tissue injury [81, 82]. IL-10 is a key anti-inflammatory cytokine that powerfully inhibits the expression of Th1 cytokines, including IL-2 and IFN-γ [53, 83], and suppresses the production of TNF-α, IL-1β, IL-6, IL-8, IL-12, GM-CSF, MIP-1α and MIP-2α by monocytes, macrophages, neutrophils and NK cells [84, 85]. Persistent IL-10 overproduction cause anergy and immunosuppression leading to reinfection and death [86, 87]. However, we found that treatment with OLDA did not impair bacterial clearance in mice (Fig. 4), which is similar to the effects of proresolving lipid mediators described by other investigators including the *n*−3 fatty acid-derived resolvins and protectins [88–92], and NADA [37, 93]. We reported that several cannabinoids including Δ9-THC, Win55,212-2, and Hu-210 induce IL-10 and downregulate pro-inflammatory cytokines in endotoxemic mice [38]. These endogenous and exogenous lipids act on cannabinoid and vanilloid receptors that are primarily expressed in the PNS and/or CNS [94]. We speculate that cannabinoids and vanilloids also act as proresolving lipid mediators, and that through their actions at their neuronal receptors, regulate systemic immune functions, and promote inflammatory resolution and the return to immune homeostasis.

The understanding of the integrative bi-directional communication between the nervous and immune system is still rudimentary [95–97]. On the one hand, cytokines and other immune factors have been reported to affect the level of activity and responsivity of discharges in sympathetic and parasympathetic nerves, which informs the CNS of the peripheral immune status [97]. On the other hand, recent studies suggest that neural innervation of primary and secondary lymphoid organs regulates inflammation [98]. Among them, the most extensively studied is the “cholinergic anti-inflammatory reflex”, which is mediated proximally by vagal efferent neuron activation and downstream by the spleen [66, 68]. This reflex involves interactions between the autonomic nervous system including neural adrenergic (sympathetic) and cholinergic (parasympathetic) pathways and conventional immune cells (T cells and macrophages) in the spleen. We found that OLDA’s anti-inflammatory effects were retained in splenectomized endotoxemic mice. This suggests that the cholinergic anti-inflammatory reflex, which requires the spleen, does not mediate the TRPV1-dependent anti-inflammatory effects of OLDA. Conceivably the downstream anti-inflammatory effects of OLDA are mediated by efferent neurons that innervate the bone marrow [99] and regulate the release of MSDCs.

There are strong links, and interplay between the endovanilloid and the endocannabinoid systems, which share ligands and regulatory enzymes [100]. The *N-*acyl-dopamines NADA and OLDA are TRPV1 agonists, and putative agonists of CB_1_R and other GPCRs [94]. The endocannabinoids 2A-G and AEA can also bind TRPV1 in addition to CB_1_R, CB_2_R and other GPCRs [94]. We reported that CB_1_R activation by Δ9-THC leads to sustained anti-inflammatory and pro-resolution responses in mice with acute inflammation. Specifically, Δ9-THC increases IL-10 secretion by monocytic myeloid-derived suppressive cells (Mo-MDSCs) in a CB_1_R-dependent manner in endotoxemic mice [38]. The pattern of immunomodulation induced by OLDA is similar to that induced by NADA [37] and Δ9-THC [38], which also upregulate IL-10 and downregulate pro-inflammatory cytokines in the blood. CB_1_R is most highly expressed in CNS neurons [12]. Based on the similarities in the anti-inflammatory effects of OLDA, NADA [37] and Δ9-THC [38] we speculate that their engagement of TRPV1 (NADA, OLDA) or CB_1_R (9-THC) proximally activates a shared downstream anti-inflammatory network that leads to peripheral upregulation of IL-10 production by monocytes/macrophages, and downregulation of multiple pro-inflammatory cytokines and chemokines.

In addition to our findings on the anti-inflammatory actions of exogenous OLDA in endotoxemic and septic mice, we found that acute inflammation induced by endotoxemia modulates levels of endocannabinoids, endovanilloids, and other lipid mediators in the brain and systemic circulation. Along with reports of similar findings in the context of traumatic brain injury [101, 102] or brain inflammation [103], our data suggest that the endovanilloid system and closely related endocannabinoid system may be dynamically regulated during sepsis and acute injuries to balance the early systemic inflammatory response. We speculate that systemic inflammation induces an anti-inflammatory reflex via the endogenous production of OLDA and NADA in the brain, which in turn acts on CNS TRPV1 neurons to upregulate the systemic production of IL-10, and limit downstream “bystander” organ injury.

## Conclusions

**Fig. 7:**
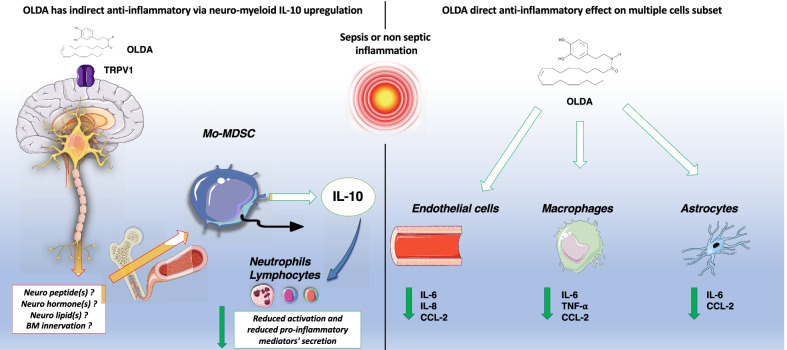
Hypothetical model of OLDA’s anti-inflammatory effects mediated by CNS TRPV1 neurons, and TRPV1-independent actions on leukocytes and endothelial cells. Left panel: Under inflammatory conditions, the activation of central nervous system TRPV1 neurons by OLDA triggers a neuro-immune inflammatory reflex, that culminates in the acute upregulation of IL-10 production in the peripheral circulation. Monocytic myeloid cells are required for the upregulation of IL-10 and are speculated to be the cellular source of the upregulated IL-10. The link between neuronal TRPV1 activation in the CNS and the effector myeloid cells remains unknown. Potential mechanistic mediators include neuromodulators released by TRPV1 neurons, or bone marrow innervation. Right panel: In addition to the neuro-immune upregulation of IL-10, OLDA acts directly upon monocytes/macrophages and/or non-conventional immune cells, such as endothelial cells and glial cells, to reduce their production of pro-inflammatory cytokines and chemokines. Therefore, the endovanilloid system has a multi-pronged ability to limit the amplification of acute inflammation and promote its resolution

Our study provides evidence of a novel multicellular network that, during acute inflammation regulates the systemic innate immune response via the activation of CNS TRPV1 neurons (Fig. 7, left panel). Our in vitro data with NADA [37] and OLDA suggest that these acyl-dopamines can also directly reduce inflammatory activation of cultured leukocytes, astrocytes and endothelial cells, but do so independently of TRPV1 (Fig. 7, right panel). The endovanilloid and endocannabinoid systems are remarkably well-preserved through evolution, and they play roles in multiple vital systems and processes, including pain, behavior and reproductive function [104, 105]. The systems are redundant and pleiotropic, sharing receptors and enzymes [106, 107]. We observed that LPS-induced inflammation modulates brain levels of multiple endovanilloids and endocannabinoids. We hypothesize that the dynamic regulation of these homeostatic systems during inflammation and sepsis lead to protective innate neuro-immune reflexes that limit systemic inflammation, promote inflammatory resolution, and ultimately protect the host from shock and organ injury. Overall, our results uncover a unique interplay between the CNS TRPV1 and raise the exciting possibility of therapeutically targeting this neuro-immune network in acute and chronic inflammatory disorders.

## Supplementary Information

**Additional file 1.** Supplentary figures and legends (Figures S1–S7).

## Data Availability

Data and material are available on reasonable request.

## Abbreviations

1-AG: 1-Arachidonoyl glycerol
12-HETE: 12-Hydroxyeicosatetraenoic acid
13-HODE: 13-Hydroxy-9Z,11E-octadecadienoic acid
15-HETE: 15-Hydroxyeicosatetraenoic acid
18-HEPE: 18-Hydroxy-5Z,8Z,11Z,14Z,16E-eicosapentaenoic acid
2-AG: 2-Arachidonoyl glycerol
2-AGE: 2-Arachidonyl glyceryl ether
20-HETE: 20-Hydroxyeicosatetraenoic acid
5-HEPE: 5-Hydroxyeicosapentaenoic acid
5-HETE: 5-Hydroxyeicosatetraenoic acid
8-HEPE: 8-Hydroxy-5Z,9E,11Z,14Z,17Z-eicosapentaenoic acid
9-HODE: 9-Hydroxyoctadecadienoic acid has
AEA: Anandamide
ALI: Acute lung injury
BAL: Broncho alveolar lavage
BMDM: Bone marrow-derived macrophages
CB_1_R: Cannabinoid receptor type 1
CNS: Central nervous system
D12-PJ2: 15-Deoxy-delta-12,14-prostaglandin J2
DEA: *N*-Docosatetraenoylethanolamine
DH-g-LEA: *N*-Dihomo-γ-linolenoylethanolamine
DRG: Dorsal root ganglion
Foxp3: Forkhead box P3
LEA: Linolenoylethanolamine
MHC: Major histocompatibility complex
MSS: Mouse sepsis score
MSSA: Methicillin-susceptible *Staphylococcus aureus*
NADA: *N*-Arachidonoyl dopamine
O-AEA: Virodhamine
OEA: Oleoylethanolamine
OLA: Oleamide
OLDA: *N*-Oleoyl dopamine
Pam3Cys: Tripalmitoyl-S-glyceryl cysteine
PNS: Peripheral nervous system
SEA: Stearoylethanolamine
TRPV1: Transient receptor potential cation channel subfamily V member 1
