# Supplementary material for: N-Oleoyl dopamine induces IL-10 via central nervous system TRPV1 and improves endotoxemia and sepsis outcomes
Source: J Neuroinflammation. 2022 May 24;19:118. doi: 10.1186/s12974-022-02485-z (PMC9131699; doi:10.1186/s12974-022-02485-z)
Supplement: Supplementary file 1 — Additional file 1. Supplentary figures and legends (Figures S1–S7). [file 12974_2022_2485_MOESM1_ESM.pdf]

Figure S1

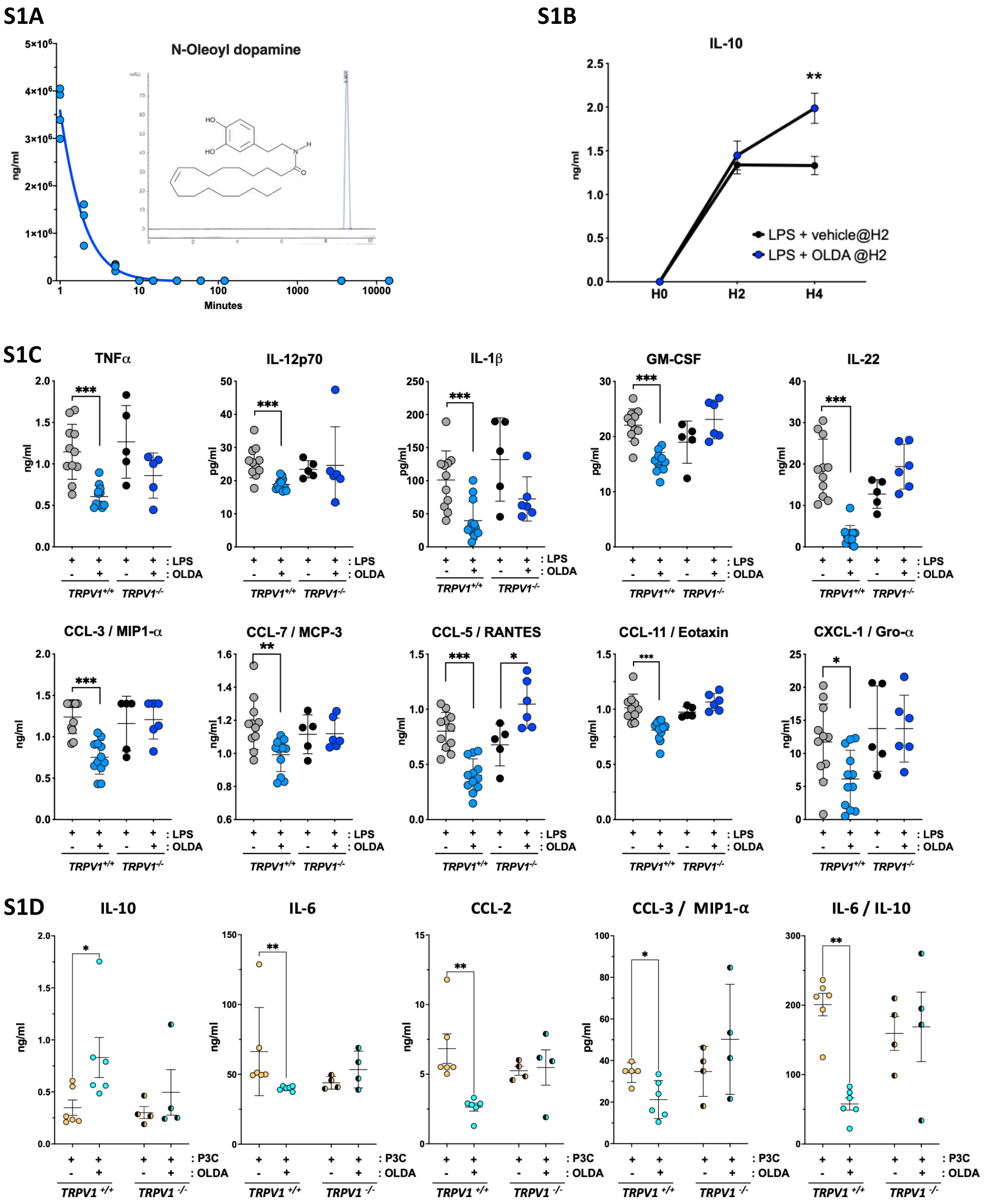

S1A
N-Oleoyl dopamine
ng/ml
Minutes
S1B
IL-10
ng/ml
LPS + vehicle@H2
LPS + OLDA @H2
H0
H2
H4
S1C
TNFα
IL-12p70
IL-1β
GM-CSF
IL-22
CCL-3 / MIP1-α
CCL-7 / MCP-3
CCL-5 / RANTES
CCL-11 / Eotaxin
CXCL-1 / Gro-α
: LPS
: OLDA
TRPV1+/+
TRPV1-/-
S1D
IL-10
IL-6
CCL-2
CCL-3 / MIP1-α
IL-6 / IL-10
: P3C
: OLDA
TRPV1 +/+
TRPV1 -/-

Figure S1 Legend:

(S1A) Pharmacokinetic study of OLDA following i.v. administration to adult wild-type mice. Plasma levels of OLDA were quantified following i.v. administration of OLDA 5 mg/kg. Plasmas were extracted using a methyl tert-butyl ether (MTBE)-based liquid-liquid method, and then underwent LC-MS/MS analysis. The concentrations are depicted as the ratio of integrated areas of the chromatographic peaks (ion count vs. time) corresponding to ΔOLDA to the integrated areas of designated internal standards. (9-week male, *n* = 4 mice per time point respectively at 1, 2, 5, 10, 15, 30, 60, 120 min, 6 hours and 24 hours after OLDA injection. **(S1B)** Post-treatment of wild-type mice with OLDA at T = 2 hours (H2) after inducing inflammation with LPS led to augmented IL-10 levels at T = 4 hours (H4) as compared with baseline prior to LPS administration (H0). (9- to 12-week female, *n* = 6-8/group). (**S1C**) The anti-inflammatory effects of OLDA were abolished in *Trpv1*$^{-/-}$ mice. Wild-type and *Trpv1*$^{-/-}$ mice were treated with LPS (1 mg/kg, i.v.) immediately thereafter were treated with OLDA (10 mg/kg, i.v.) or vehicle (i.v.). Cytokines and chemokines were quantified in plasma at 2 hours. (9- to 12-week male and female, *n* = 5-11/group). (**S1D**) OLDA reduced circulating IL-6, CCL2, and increased IL-10 levels in mice challenged with a TLR1/2 agonist in wild-type but not *Trpv1*$^{-/-}$ mice. Wild-type and *Trpv1*$^{-/-}$ mice were treated with tripalmitoyl-S-glyceryl cysteine (P3C) (1 mg/kg, i.v.) and immediately thereafter with OLDA (10 mg/kg, i.v.) or vehicle (i.v.). IL-10, IL-6, and CCL2 were quantified in plasma at 2 hours (11-week male, *n* = 4-6/group). * $P < 0.05$, ** $P < 0.01$ *** $P < 0.001$, two-tailed Mann-Whitney *U* test.

Figure S2

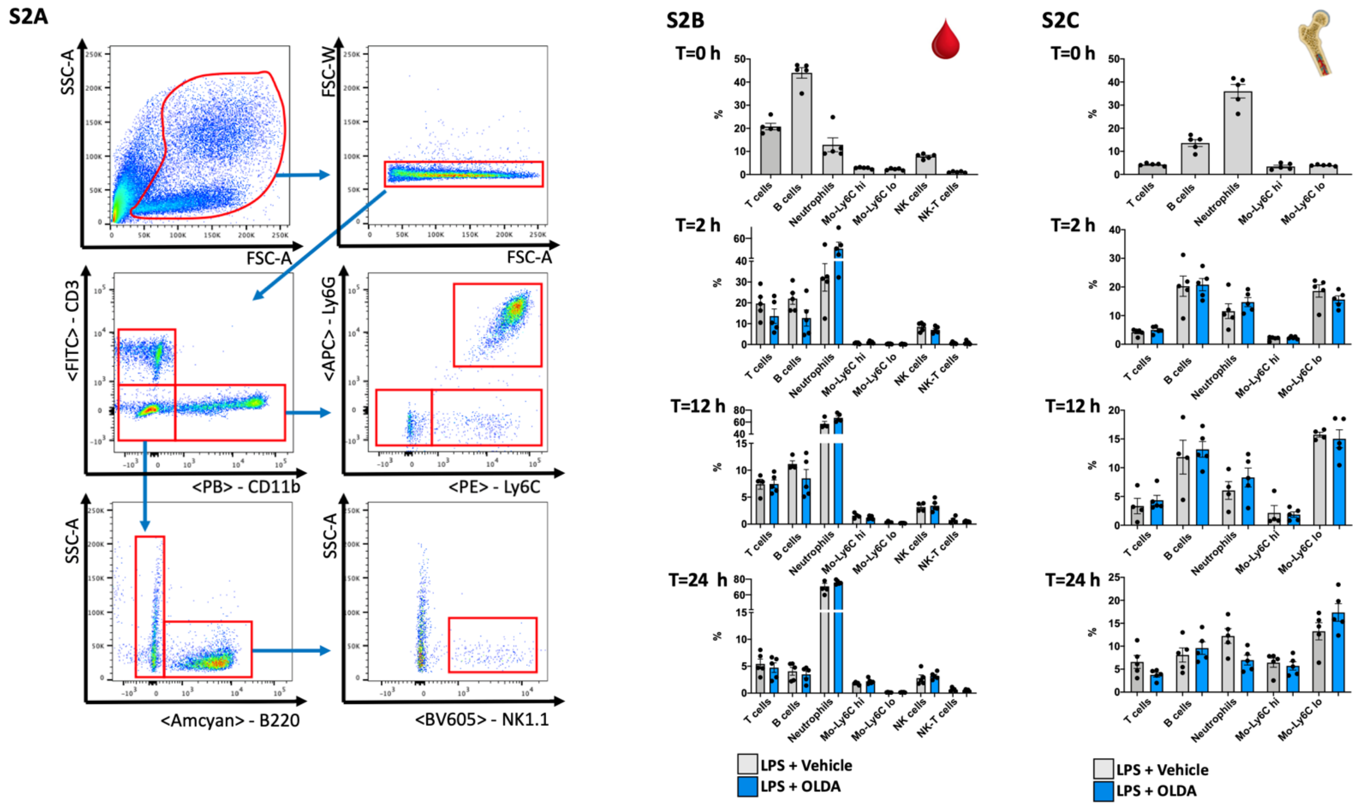

S2A
SSC-A
FSC-A
FSC-W
FSC-A
<FITC> - CD3
<PB> - CD11b
<APC> - Ly6G
<PE> - Ly6C
SSC-A
<Amcyan> - B220
SSC-A
<BV605> - NK1.1
S2B
T=0 h
T=2 h
T=12 h
T=24 h
%
T cells
B cells
Neutrophils
Mo-Ly6C hi
Mo-Ly6C lo
NK cells
NK-T cells
LPS + Vehicle
LPS + OLDA
S2C
T=0 h
T=2 h
T=12 h
T=24 h
%
T cells
B cells
Neutrophils
Mo-Ly6C hi
Mo-Ly6C lo
LPS + Vehicle
LPS + OLDA

Figure S2 Legend:

(S2A) Flow cytometry gating strategy applied in blood and bone marrow. (**S2B-C**) Dynamics of leukocyte subsets in the blood (**S2B**) and bone marrow (BM) (**S2C**) in mice treated with LPS (3 mg/kg, i.v.) followed immediately by vehicle (Grey bar) or OLDA (10 mg/kg, i.v.) (Blue bar), analyzed at T = 0 (saline), T = 2, T = 12, and T = 24 hours. (12-week male, *n* = 5 mice per group per time point).

Figure S3

S3A

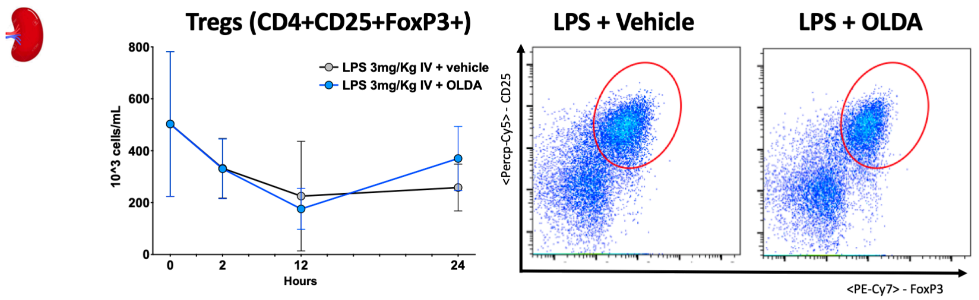

S3B

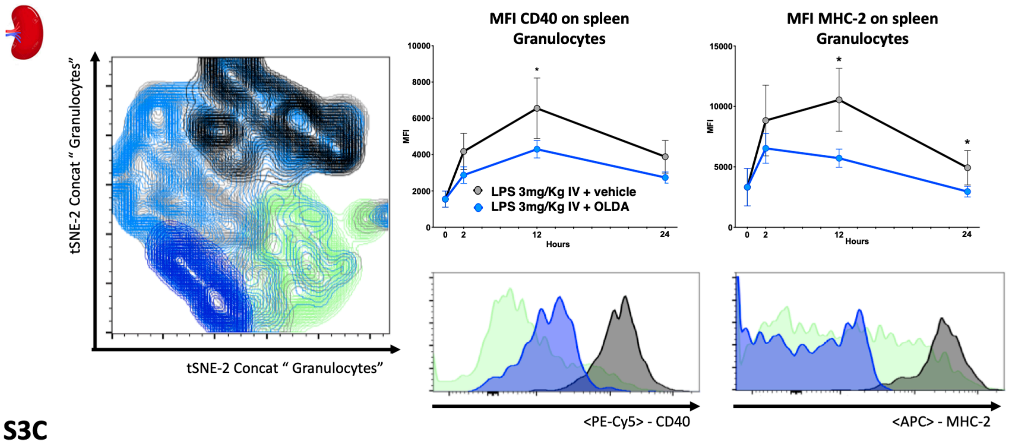

S3C

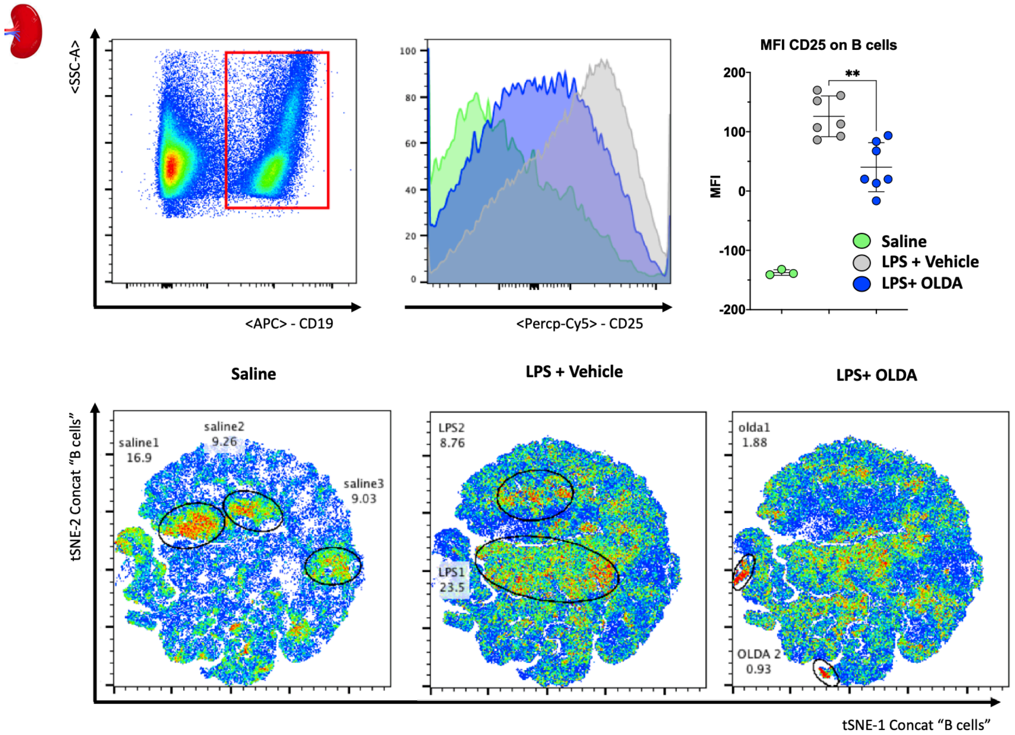

Figure S3 Legend:

(**S3A**) OLDA treatment did not affect spleen Tregs (gated as CD4+CD25+ FoxP3+) lymphocytes count, during endotoxemic shock. Wild-type mice were challenged with LPS (3 mg/kg, i.v.) and immediately thereafter were treated with OLDA (10 mg/kg, i.v.) or vehicle (i.v.) and euthanized T = 0 (saline), T = 2, T = 12, and T = 24 hours. Representative illustrations shown are at 12 hours (12-week-old male, n = 5 per group per time point). **(S3B)** OLDA reduced activation marker expression on spleen neutrophils (gated as CD11b hi, Ly6G hi) characterized by reduced expression of MHC2 and CD40 at T = 12 and 24 hours in OLDA-treated endotoxemic mice. Illustrations of concatenated sample for each group are shown at 12 hours post-LPS injection. (12 weeks old male, n = 5 per group per time point). **(S3C)** OLDA treatment reduced spleen B cell activation, as defined by CD25 expression (MFI). (8-week male, n = 7/group LPS + Vehicle and LPS + OLDA, n = 3 for saline controls). Representative illustrations shown are at 24 hours. t-SNE plot were generated using concatenated sample for each condition: It included <IgM, CD21, CD23, CD5, CD1d, CD25> for CD19+ B cells. * $P < 0.05$, ** $P < 0.01$ ***, two-tailed Mann-Whitney U test.

Figure S4

S4A

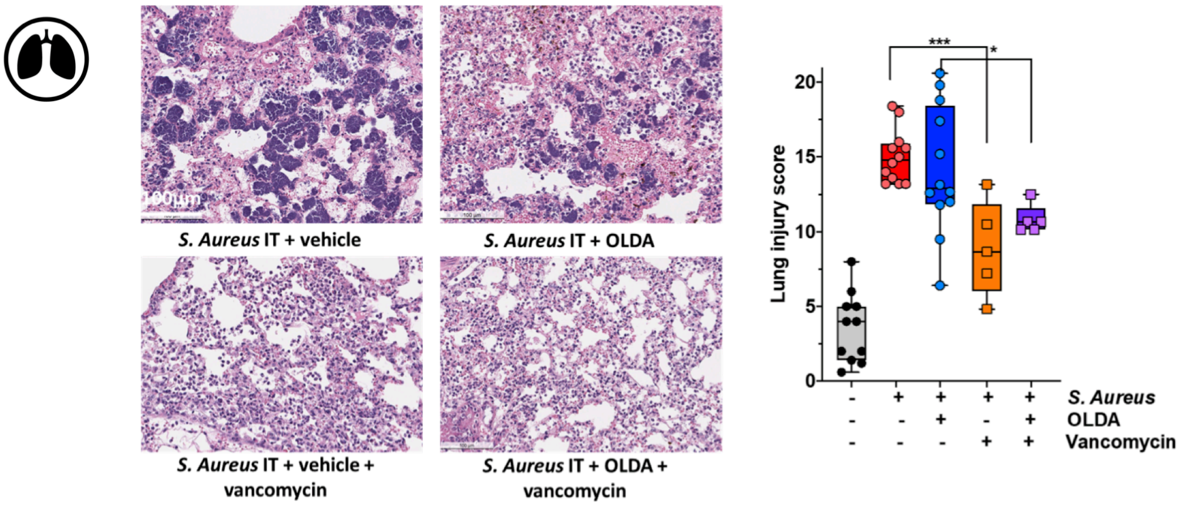

S4B

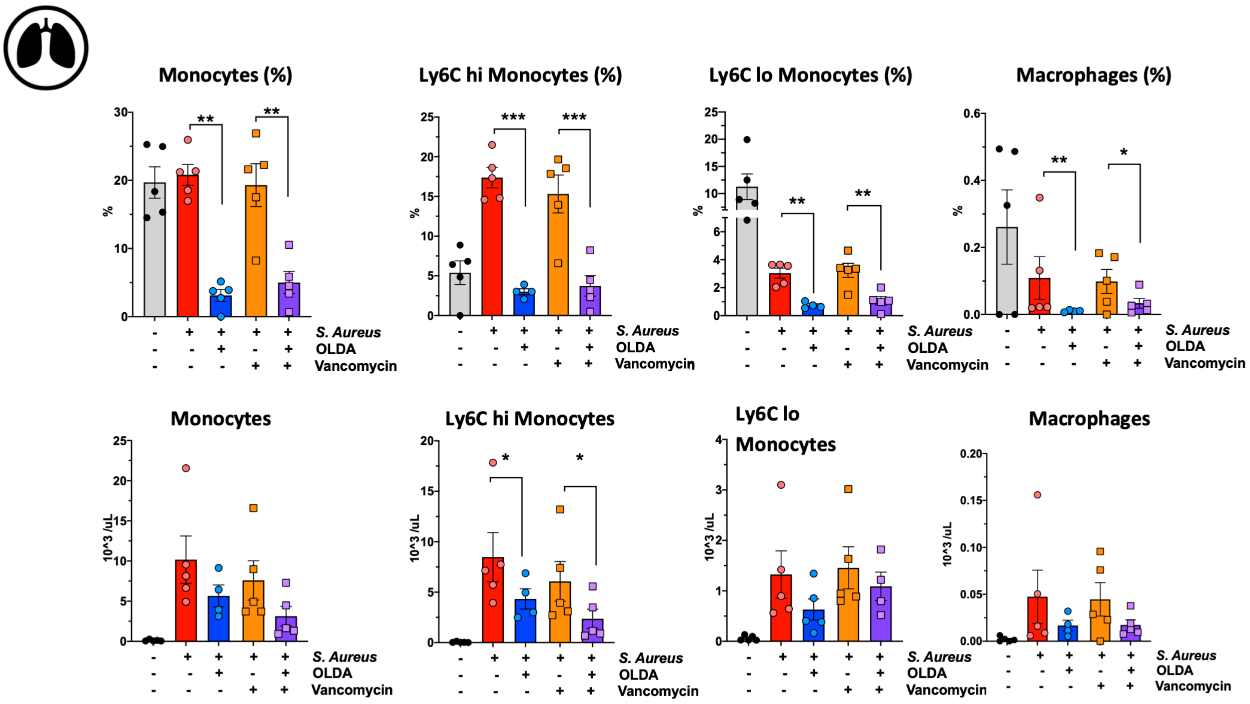

Figure S4 Legend:

(S4A) Representative H&E-stained sections of lungs (original magnification X20) collected from mice 24 hours after *S. aureus* challenge. OLDA treatment did not reduce histologic evidence of *S. aureus*-induced lung injury. Vancomycin treatment significantly reduced anatomical patterns of lung injury (10- to 12-week-old male, n = 12/group for mice that did not receive vancomycin, n = 5/group for mice that did not receive vancomycin). (**S4B**) OLDA treatment reduced recruitment of both Ly6Chi and Ly6Clo monocytes in the lung during *S. aureus* pneumopathy. These immunological effects were observed independently of vancomycin treatment. Representative illustrations shown are at 24 hours (10- to 12-week-old male, n = 4-5/group). * $P < 0.05$, ** $P < 0.01$ *** $P < 0.001$, two-tailed Mann-Whitney U test.

Figure S5

S5A

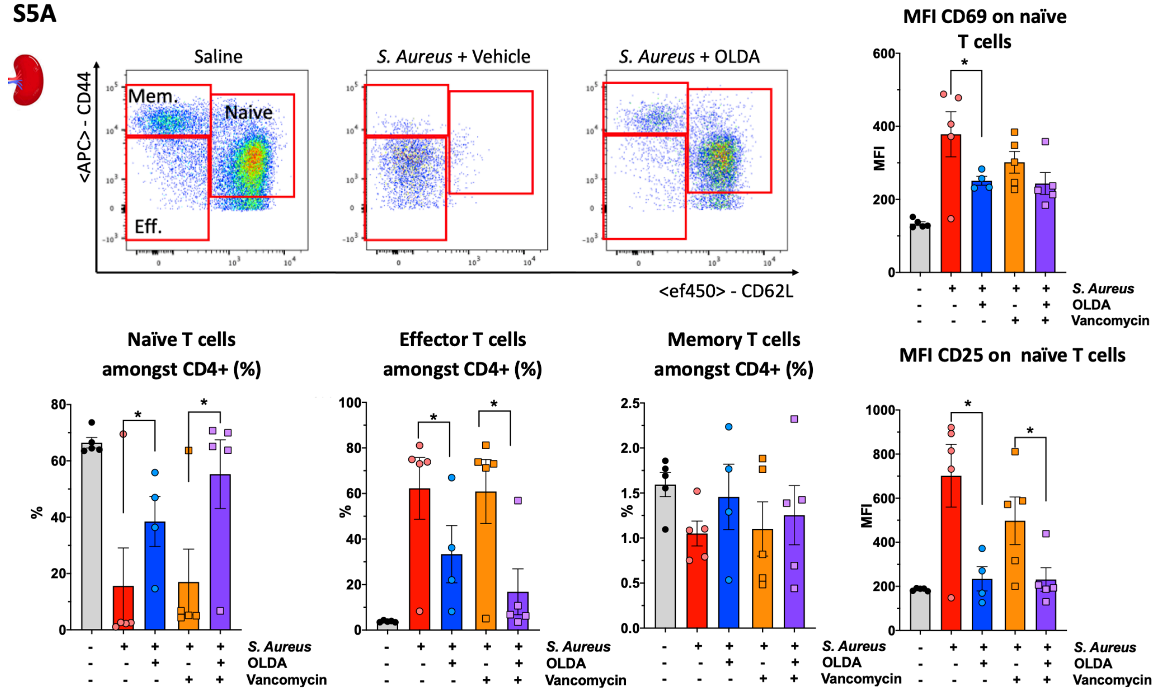

S5B

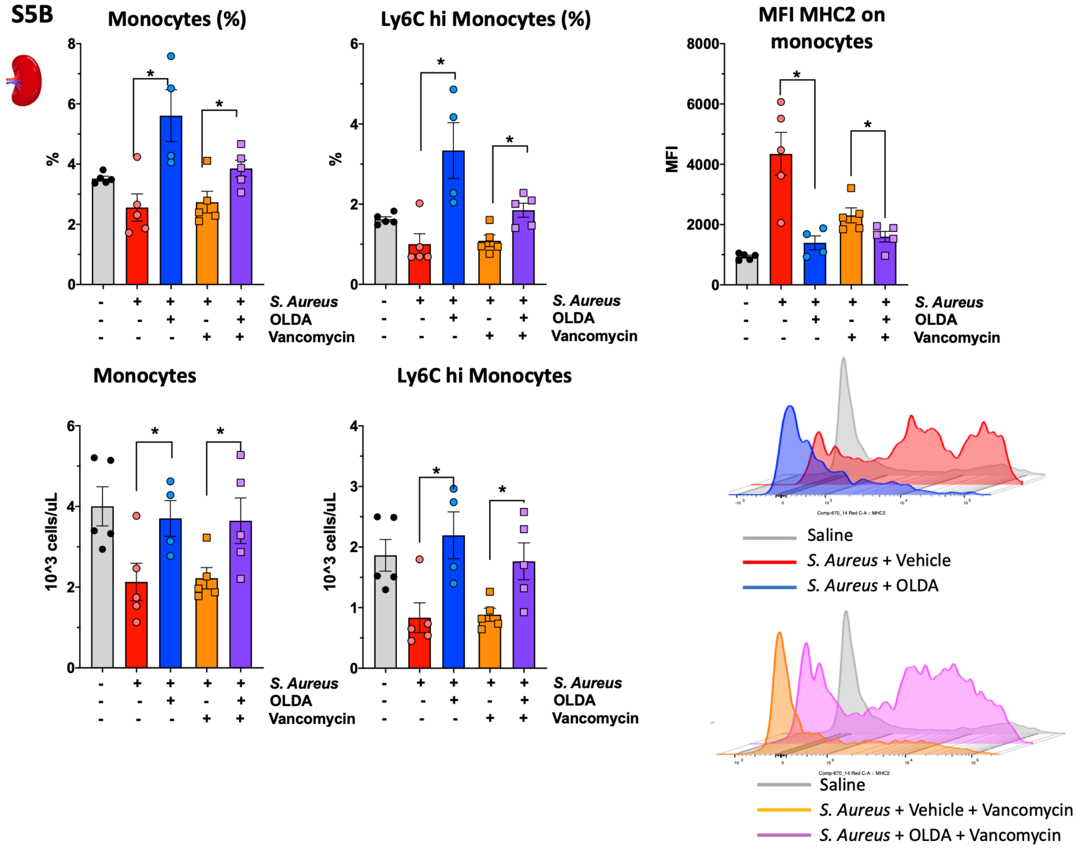

Figure S5 Legend:

(S5A) OLDA treatment reduced spleen T cell activation, defined as reduced % of effector T cells (CD44-CD62L-) amongst CD4+ T cells, and reduced CD25 and CD69 expression on naïve CD4+ T cells (CD44+ CD62L+) during *S. aureus* pneumopathy. **(S5B)** OLDA treatment reduced MHC2 expression in spleen monocytes and reduces Ly6Chi monocytes mobilization from the spleen. These immunological effects were independent of antibiotic treatment (Vancomycin). Representative illustrations shown are at 24 hours. (12-week-old male, n = 4-5/group for all panels). * $P < 0.05$, two-tailed Mann-Whitney U test.

Figure S6

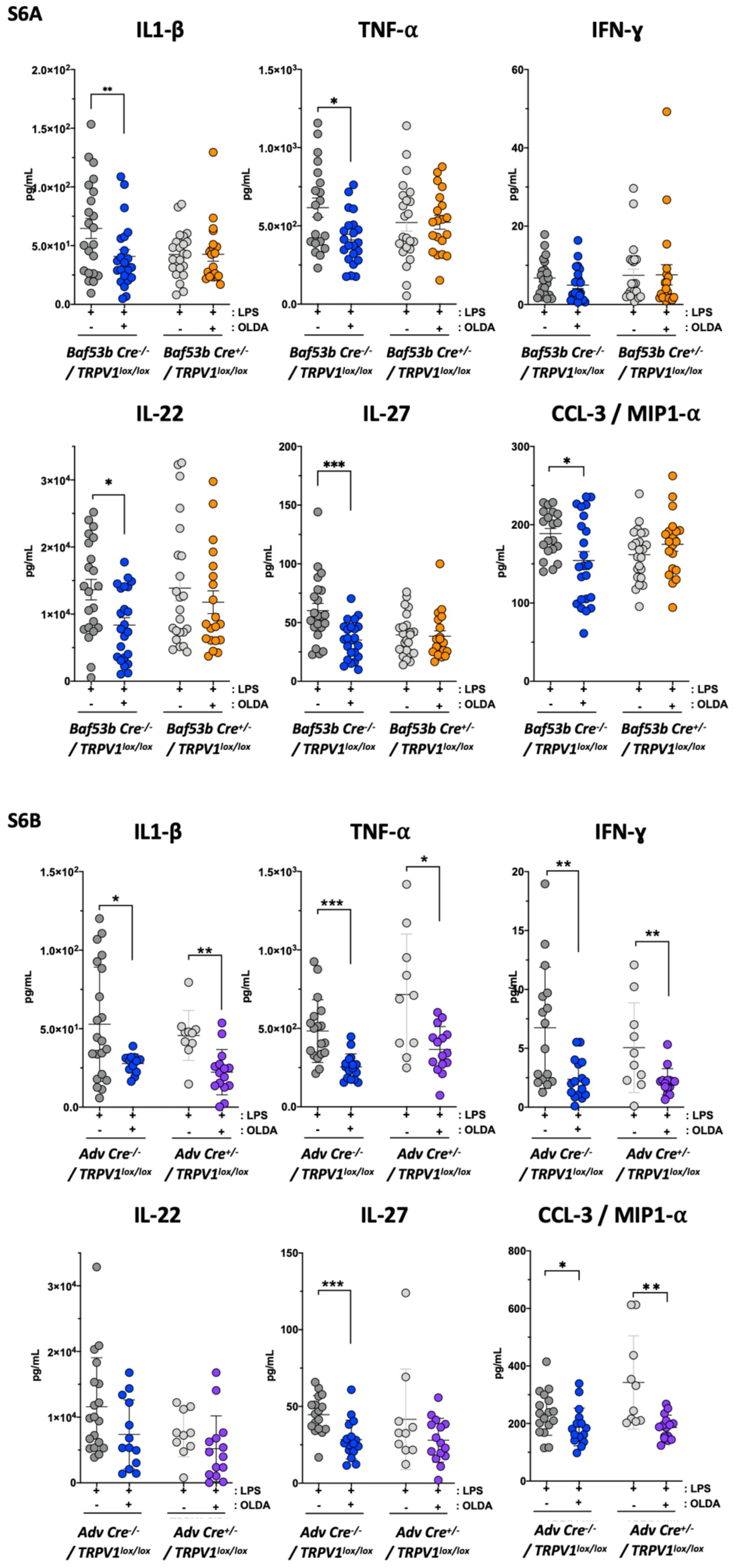
S6A
IL1-β
TNF-α
IFN-γ
IL-22
IL-27
CCL-3 / MIP1-α
pg/mL
: LPS
: OLDA
Baf53b Cre-/- / TRPV1lox/lox
Baf53b Cre+/- / TRPV1lox/lox
S6B
IL1-β
TNF-α
IFN-γ
IL-22
IL-27
CCL-3 / MIP1-α
Adv Cre-/- / TRPV1lox/lox
Adv Cre+/- / TRPV1lox/lox

Figure S6 Legend:

(S6A) Pan-neuronal TRPV1 deficiency abrogated most of the OLDA-induced down-regulation of circulating pro-inflammatory mediators in endotoxemic mice. Baf53b Cre$^{-/-}$/ *Trpv1*$^{lox/lox}$ and Baf53b Cre$^{+/-}$/ *Trpv1*$^{lox/lox}$ mice were treated with LPS (1 mg/kg, i.v.) and OLDA (10 mg/kg i.v.) or vehicle (i.v.). Plasma was collected at T = 2 hours. (9- to 12-week male and female, n = 20-22/group). **(S6B)** Specific sensory neurons TRPV1 deficiency did not affect OLDA's anti-inflammatory effect in LPS-induced in endotoxemia. Adv Cre$^{-/-}$/ *Trpv1*$^{lox/lox}$ and Adv Cre$^{+/-}$/ *Trpv1*$^{lox/lox}$ were stimulated with LPS (1 mg/kg, i.v.) and OLDA (10 mg/kg i.v.) or vehicle (i.v.). Plasma was collected at 2 hours. (8- to 12-week male and female, n = 10-21/group). * $P < 0.05$, ** $P < 0.01$ *** $P < 0.001$, two-tailed Mann-Whitney U test.

Figure S7

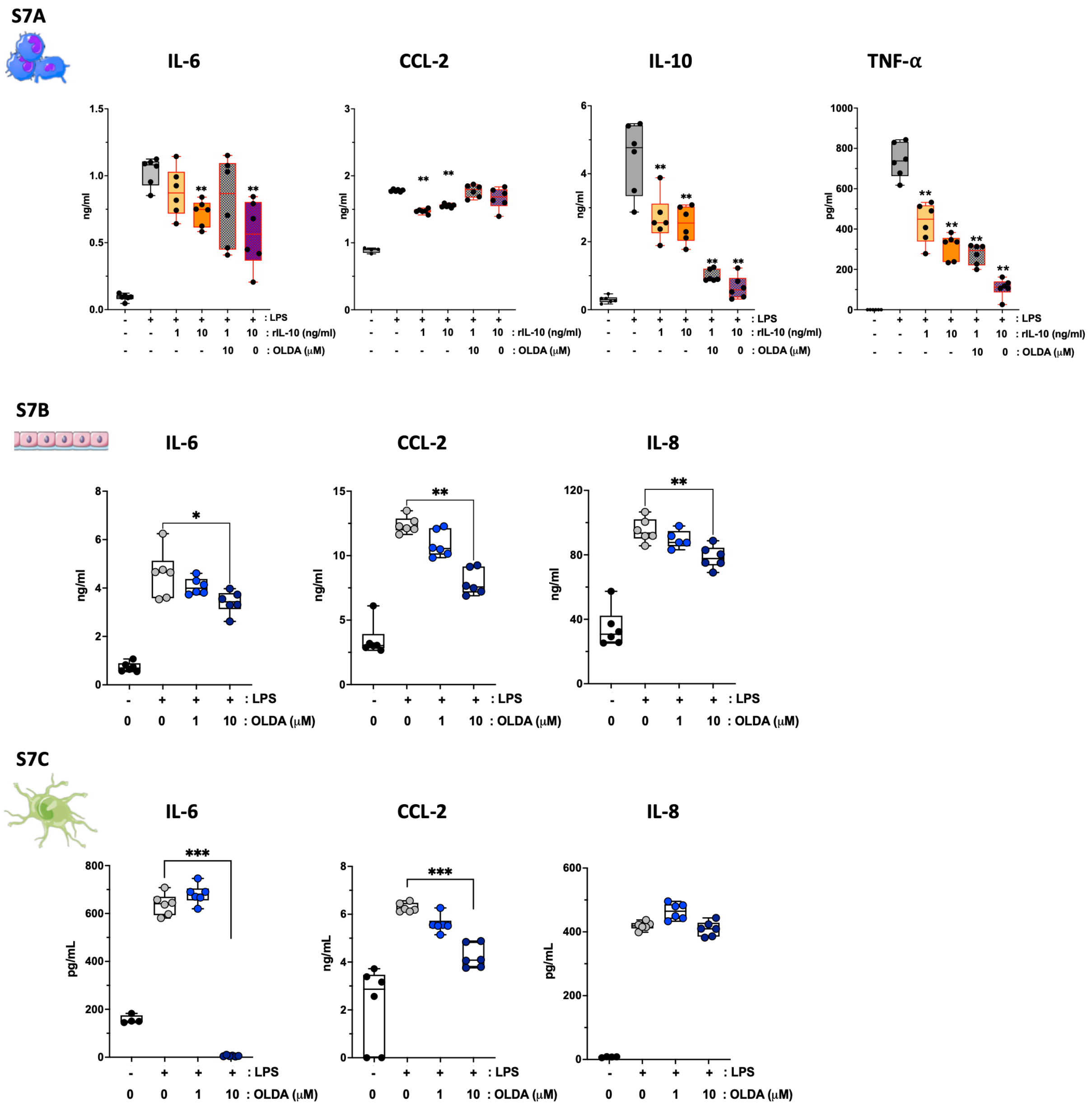

S7A
IL-6
CCL-2
IL-10
TNF-α
ng/ml
pg/ml
: LPS
: rIL-10 (ng/ml)
: OLDA (μM)
S7B
IL-6
CCL-2
IL-8
ng/ml
S7C
IL-6
CCL-2
IL-8
pg/mL
ng/mL
: LPS
: OLDA (μM)

Figure S7 Legend:

(**S7A**) *Ex vivo*, OLDA and IL-10 (human recombinant) had additive effects on reducing the LPS-induced pro-inflammatory cytokines (IL-6, CCL2 and TNF-α) and IL-10 secretion by BMDM from wild-type mice. (**S7B-C**) OLDA reduces LPS-induced pro-inflammatory mediator secretion by **(S7B)** microvascular endothelial cells (human lung MVEC) (IL-6, CCL2 and IL-8), and **(S7C)** astrocytes (NHA) (IL-6 and CCL2). Cells were treated simultaneously with LPS (1 μg/ml) +/- OLDA (1 to 10 μM) +/- human rIL-10 (1 to 10 ng/mL) (n = 6 replicates/condition). Cytokines were assayed in the supernatant after 48 hours (BMDM) or 24 hours (MVEC and NHA). * $P < 0.05$, ** $P < 0.01$ *** $P < 0.001$, two-tailed Mann-Whitney U test.
